# Incomplete climate‐driven peripatric speciation in *Moehringia* sect. *Moehringia* (Caryophyllaceae) in the European Alps

**DOI:** 10.1002/ajb2.70252

**Published:** 2026-08-26

**Authors:** Joachim W. Kadereit, Kirra McLeod, Audie M. Putra, Gudrun Kadereit, Diego F. Morales‐Briones

**Affiliations:** ^1^ Princess Therese von Bayern Chair of Systematics, Biodiversity and Evolution of Plants, Ludwig‐Maximilians‐Universität München Menzinger Str. 67 80638 Munich Germany

**Keywords:** Caryophyllaceae, European Alps, habitat divergence, *Moehringia*, peripatric speciation, Quaternary

## Abstract

**Premise:**

The origin of endemic species in the European Alps is commonly attributed to the climatic oscillations of the Quaternary. *Moehringia* sect. *Moehringia*, with 12 of 15 species endemic to the Alps and mostly restricted to well‐known glacial refugia, is a prime system for investigating the diversification of a lineage originating in the Alps.

**Methods:**

We used all species in this lineage, most of which were represented by multiple accessions, and a target enrichment approach with baits specifically designed for *Moehringia* to obtain a phylogenetic tree from 829 nuclear loci. We also dated the phylogenetic tree with a molecular clock approach.

**Results:**

Most species originated in the Quaternary. Four regionally distributed species (*M. intermedia*, *M. markgrafii*, *M. papulosa*, *M. tommasinii*), all nested within the widespread *M. muscosa*, were found to be between 1.36 and 0.25 million years old. *Moehringia muscosa* and the four species derived from it differ in habitat, leaf succulence, and glaucousness.

**Conclusions:**

Because the widespread species has not coalesced into monophyly and the regionally distributed species have evolved in glacial refugia, we consider speciation in this lineage incomplete and climate‐driven. Divergence in habitat and leaf morphology between the widespread and the derived species suggests a role of adaptive evolution.

Based on numerous dated molecular phylogenetic trees, it is well established that much of plant speciation worldwide occurred in the Quaternary, i.e., the last 2.6 million years (reviewed by Kadereit and Abbott, 2022). This speciation was most likely driven by the strong climatic oscillations of the Quaternary, which led to changes in geographical distributions (latitudinal, longitudinal, and elevational) due to variations in temperature, precipitation, and sea level. Changes in geographical distribution often led to the fragmentation of distribution ranges, providing opportunities for divergence and speciation in geographical isolation. Genetic drift in small, isolated populations and adaptive evolution in response to changing abiotic and biotic conditions may have contributed to evolutionary divergence. The relative importance for Quaternary speciation in isolated populations of these factors, i.e., genetic drift in small populations and natural selection, or their combination, has been assessed differently by different authors (Hewitt, 1996, 2000, 2004; Tribsch and Schönswetter, 2003; Tribsch, 2004; Hampe and Petit, 2005; Carstens and Knowles, 2007; Casazza et al., 2008b, 2010, 2016; Stewart et al., 2009; Hampe and Jump, 2011; Stewart and Stringer, 2012; Mee and Moore, 2014; Woolbright et al., 2014; Gentili et al., 2015a, b; Kiedrzyński et al., 2017; Kadereit, 2023, 2024).

The European Alps, a region of high species diversity per unit area (Barthlott et al., 1996), harbor approximately 4000 native vascular plant taxa (species and subspecies), of which approximately 400 are considered endemic to the area (Aeschimann et al., 2004). According to Tribsch and Schönswetter (2003), the distribution of endemic species in the Alps was first related to the location of glacial refugia by de Candolle (1875) and Chodat and Pampanini (1902). As a result of the more or less continuous ice cover of the Alps in Quaternary glacials, best documented for the Last Glacial Maximum, most refugial areas, identified by the concentration of endemic species in combination with geomorphological evidence, are located on the edge of the Alps (Tribsch and Schönswetter, 2003; Schönswetter et al., 2005). It has commonly been assumed that the distribution of endemic species in glacial refugia and their evolutionary divergence and eventual speciation are causally linked, and that, as outlined above, genetic drift in small, geographically isolated populations, adaptive evolution, or their combination resulted in speciation.

While it remains unclear which forces drove the evolutionary divergence of refugial populations, it is also unclear whether such speciation resulted in the extinction of the progenitor species. On the one hand, it is conceivable that a progenitor species was pushed into a refugial area by advancing glaciers and a changing climate, where it evolved into what today is the refugial endemic. This scenario implies extinction of the progenitor. On the other hand, a progenitor species might have been pushed into different refugial areas, in some of which evolutionary change and speciation took place, but not in others. In such a scenario, the progenitor of refugial endemics might still be extant, and a pattern of allo‐ or peripatric speciation might be recognizable.

*Moehringia* L. (Caryophyllaceae) comprises two clades, sect. *Moehringia* and sect. *Latifoliae* Nyman ex Graebn. in Asch. & Graebn. (Fior and Karis, 2007). Section *Moehringia*, with 15 perennial herbaceous species (Kadereit, 2025), is notable for its high number of endemic species in the European Alps. These 15 species have very different distributions, with two species being relatively widespread and 13 being regionally or locally distributed (Figure 1). *Moehringia ciliata* (Scop.). Dalla Torre is widespread across the Alps with scattered outposts on the western Balkan Peninsula (Friedrich, 1969; Hind, 1988, 1993), and *M. muscosa* L. is widely distributed in the Alps but also grows at higher elevations in southwestern, southern, and southeastern Europe (Figure 1; Friedrich, 1969; Hind, 1988, 1993). Of the more regionally distributed species, *M. bavarica* (L.) Gren. consists of one subspecies (subsp. *bavarica*) of limited distribution in the Alps (Figure 1) and one subspecies (subsp. *malyi* (Hayek) Kadereit) of regional distribution in the Alps (Styria/Austria) but also with a wide range of very scattered occurrences on the Balkan Peninsula (for detailed distribution see Sauer, 1965; Kadereit, 2025). The remaining 12 species are mostly regional or local endemics along the southern edge of the Alps (nine species) or in geographically adjacent areas (three species; Figure 1). The distribution of endemics in *Moehringia* sect. *Moehringia* illustrates that endemics are not evenly distributed across the Alps (de Candolle, 1875; Chodat and Pampanini, 1902; Pawłowski, 1970; Favarger, 1972; Tribsch and Schönswetter, 2003; Tribsch, 2004; Aeschimann et al., 2011; Taberlet et al., 2012; Kadereit, 2024) but that a much higher number of endemics is found along the southern than along most of the northern edge of the Alps. The greater concentration of endemic species along the southern edge of the Alps has been related to the different properties of glacial refugia north and south of the Alps (Kadereit, 2024).

**Figure 1:**
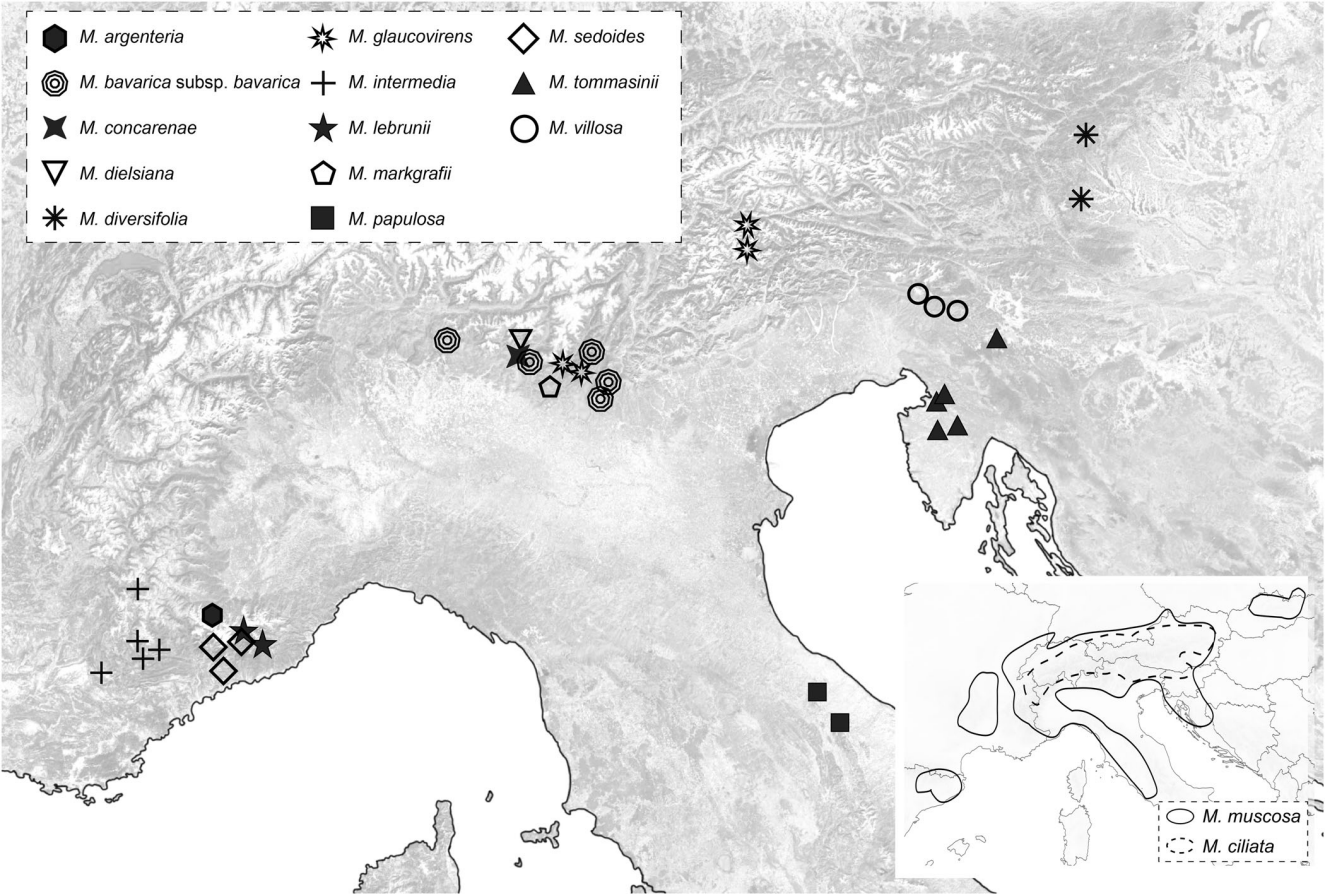
Geographical distribution of the 13 species of *Moehringia* sect. *Moehringia* regionally distributed in the Alps and adjacent areas. Inset: distribution of the widespread *M. ciliata* and *M. muscosa*.

The regional endemics of *Moehringia* sect. *Moehringia* had already attracted the attention of botanists in the past (Mattfeld, 1925; Merxmüller and Gutermann, 1957; Pitschmann and Reisigl, 1959; Minuto et al., 2006; Casazza et al., 2008b), and their morphology, distribution, and ecology are well studied (Friedrich, 1969; Hind, 1988, 1993; Fior and Karis, 2007; Landolt et al., 2010; Minuto et al., 2011; Table 1). The seeds of all species have fleshy appendages (Minuto et al., 2011), and at least those species growing in rock crevices are ant‐dispersed (Casazza et al., 2008a). Except for *M. argenteria* Casazza & Minuto, *M. concarenae* F. Fen. & F. Martini and *M. villosa* (Wulfen) Fenzl, for which chromosome numbers have not yet been determined, all species of sect. *Moehringia* have 2*n* = 24 chromosomes (Hind, 1993). Species limits in the section are uncontroversial (Friedrich, 1969; Hind, 1988, 1993; Fior and Karis, 2007; Minuto et al., 2011).

**Table 1:** Ecological and morphological characteristics of species of *Moehringia* sect. *Moehringia*. Based on Merxmüller and Gutermann (1957), Pitschmann and Reisigl (1959), Sauer (1965), Merxmüller and Grau (1967), Friedrich (1969), Zimmermann (1976), Hind (1988, 1993), Martini (1990), Fenaroli and Martini (1992), Minuto et al. (2006), Casazza and Minuto (2008), Landolt et al. (2010), Soriano et al. (2012), Fišer et al. (2014), and Dakskobler and Martinčic (2021). For *M. argenteria*, *M. tommasinii*, and *M. papulosa*, no information on soil humidity is given by Landolt et al. (2010). The information given for *M. tommasinii* is based on Martini (1990) and Fišer et al. (2014).

Species	Leaf succulence	Leaf glaucousness	Elevation (m a.s.l.)	Soil humidity	Habitat
*M. argenteria*	slightly succulent	not glaucous	>2600	—	subvertical cliffs
*M. bavarica* subsp. *bavarica*	succulent	glaucous	<1200	dry to moderately dry	vertical or overhanging cliffs
*M. bavarica* subsp. *malyi*	slightly succulent	glaucous	400–1400	dry to moderately dry	vertical or overhanging cliffs
*M. ciliata*	slightly succulent	not glaucous	1600–2700	humid	humid scree, rarely cliffs
*M. concarenae*	slightly succulent	not glaucous	(1800)1900 –2400	dry	scree
*M. dielsiana*	succulent	glaucous	1300–1400	moderately dry	vertical cliffs
*M. diversifolia*	not succulent	not glaucous	400–1800	fresh	humid and shaded gorges
*M. glaucovirens*	slightly succulent	glaucous	700–2200	fresh	steep cliffs
*M. intermedia*	slightly succulent	slightly glaucous	600–1000 m	fresh	cliffs, often shaded
*M. lebrunii*	succulent	glaucous	830–1680	moderately dry	overhanging cliffs
*M. markgrafii*	succulent	not glaucous	c. 300	moderately dry	steep cliffs
*M. muscosa*	not succulent	not glaucous	500–2300	humid	damp soils, shaded
*M. papulosa*	succulent	glaucous	c. 200	—	overhanging cliffs
*M. sedoides*	succulent	glaucous	220–2200	fresh	overhanging cliffs
*M. tommasinii*	succulent	glaucous	100–350	moderately dry	vertical to subvertical cliffs
*M. villosa*	slightly succulent	not glaucous	<1600	moderately dry	vertical cliffs, rarely scree

As one of the most species‐rich lineages endemic to the Alps (Kadereit, 2017), *Moehringia* sect. *Moehringia* offers an opportunity to study in detail the timing, geographical setting, and ecology of its diversification. Although other plant radiations centered in the Alps have been examined with similar aims (e.g., *Androsace* L.: Schneeweiss et al., 2004; Boucher et al., 2016b; *Facchinia* Rchb.: Dillenberger and Kadereit, 2017; *Phyteuma* L.: Schneeweiss et al., 2013; *Primula* L. sect. *Auricula* Duby: Zhang et al., 2004, Boucher et al., 2016a, b; *Saxifraga* Tourn. ex L. subsect. *Arachnoideae* (Engl. & Irmsch.) Tkach, Röser & M.H. Hoffm.: Gerschwitz‐Eidt et al., 2023; *Soldanella* L.: Boucher et al., 2016b; Slovák et al., 2023), in most cases, the resolution of interspecific relationships was insufficient for a full understanding of the diversification of these groups.

Using a complete species sample and a target enrichment approach with baits specifically designed for *Moehringia*, we aimed to obtain a fully resolved, well‐supported phylogenetic tree of *Moehringia* sect. *Moehringia* to understand the diversification of this lineage. In particular, we investigated whether (1) this lineage diversified in the Quaternary and (2) whether the two widespread species, *M. ciliata* and *M. muscosa*, gave rise to at least some of the many endemics. This latter hypothesis, which implies that the widespread progenitors of the endemics are still extant, is derived both from the model of Quaternary speciation of refugial endemics in the Alps, i.e., divergence and speciation in peripheral and (generally) southern refugia, and from the observation that of the 13 narrowly distributed species, seven have pentamerous flowers, as does *M. ciliata*, and six have tetramerous flowers, as does *M. muscosa*. Finally, we also (3) discuss whether the morphology and ecology of the endemics can be interpreted to indicate adaptation to changing conditions in their refugial ranges.

## MATERIALS AND METHODS

### Nuclear tree‐based orthology loci identification for target enrichment bait design

We inferred orthologous loci across Caryophyllaceae using publicly available transcriptome and genome data. We used transcriptome assemblies and their corresponding translations into coding sequences (CDSs) and outgroup reference genome CDSs from Yang et al. (2018). Additionally, we included two newly sequenced transcriptomes from *Moehringia ciliata* and *M. muscosa* (Appendix S1: Table S1). We extracted total RNA from leaves using the Qiagen RNEasy Plant Mini Kit (Valencia, CA, USA). Library preparation and sequencing were performed on an Illumina NovaSeq X Plus (PE 150) platform at Novogene Co. (Cambridge, UK). We performed read processing, transcriptome assembly, and translation of newly sequenced transcriptomes as described by Morales‐Briones et al. (2021). We corrected sequencing errors in the raw reads using Rcorrector (Song and Florea, 2015) and removed reads flagged as uncorrectable. We removed sequencing adapters and low‐quality bases with Trimmomatic 0.39 (Bolger et al., 2014). Additionally, we filtered plastid and mitochondrial reads with Bowtie 2 version 2.5.2 (Langmead and Salzberg, 2012) using publicly available Caryophyllales organelle genomes from the Organelle Genome Resources database (RefSeq; [Pruitt et al., 2007]; last accessed on 17 October 2018) as references. We discarded overrepresented sequences detected with FastQC 0.12.1 (Andrews, 2010). We performed de novo assembly using Trinity 2.15.1 (Grabherr et al., 2011) with default settings, excluding in silico normalization. We translated transcripts using TransDecoder 5.3.0 (Haas et al., 2013) with default settings, and we used the proteomes of *Arabidopsis thaliana* (L.) Heynh., *Dianthus caryophyllus* L., and *Silene latifolia* Poir. to identify open reading frames. Finally, we further reduced redundancy in CDSs from translated amino acid sequences using CD‐HIT 4.7 (‐c 0.99; [Fu et al., 2012]).

We also included the *Silene latifolia* genome (Yue et al., 2023) to facilitate exon selection for bait design. In total, we included 22 species of Caryophyllaceae and three outgroups (Appendix S1: Table S1). We inferred homology on CDSs using reciprocal BLASTN searches, followed by orthology inference using the tree‐based monophyletic outgroup (MO) approach from Yang and Smith (2014) and the methods of Morales‐Briones et al. (2021) with modifications. We carried out BLASTN searches with an *E‐*value cutoff of 10 and max_target_seqs set to 100, and filtered BLASTN hits with a minimum fraction of 0.4. We clustered putative homologs with MCL 14‐137 (van Dongen, 2000) with a minimum minus log‐transformed *E*‐value cutoff of five and an inflation value of 1.4 and retained only clusters with at least 19 taxa. We aligned individual homolog clusters using the OMM_MACSE 12.01 pipeline (Scornavacca et al., 2019) and cleaned aligned columns to have no more than 90% missing data using the function pxclsq 1.3.2 from phyx (Brown et al., 2017). We inferred homolog trees using RAxML 8.2.12 (Stamatakis, 2014) with a GTRCAT model and no node support. We pruned monophyletic and paraphyletic tips from the same species from the homolog trees by keeping only the tip with the highest number of characters in the trimmed alignment. After visual inspection of ca. 25 homolog trees, we determined that internal branches longer than 0.2 were likely representing deep paralogs. These branches were cut apart, leaving subclades with at least 19 taxa. Finally, we detected and removed abnormally long tips using TreeShrink 1.3.9 (Mai and Mirarab, 2018) with the per‐gene mode and a false positive error rate threshold (*α*) of 0.05 while excluding the outgroups from being removed. We inferred orthology using only ortholog groups with at least 19 taxa, with all Caryophyllaceae species designated as ingroups and *Beta vulgaris* L., *Spinacia oleracea* L. (both Amaranthaceae), and *Phaulothamnus spinescens* A. Gray (Achatocarpaceae) as outgroups. The MO approach filters for clusters with outgroup taxa that are monophyletic and single‐copy, filtering for single‐ and low‐copy loci. It then roots the tree using the outgroups, traverses the rooted tree from root to tip, and retains only the branch with more taxa when gene duplication is detected. When no taxon duplication was detected, we set the MO approach to output a one‐to‐one ortholog, provided that the outgroup taxa are monophyletic and single‐copy. We obtained a total of 7591 orthologous loci across Caryophyllaceae, including 3529 one‐to‐one orthologs.

### Exon selection for target enrichment bait design

Exon selection and filtering followed the methods of Kiedaisch et al. (2025) with modifications. First, we generated FASTA files for all orthologs and retained only those that included the *Silene latifolia* genome sample, resulting in 6963 orthologs. Then we hard‐masked the introns from the original *Silene latifolia* genome using BEDTools 2.30.0 (Quinlan and Hall, 2010). We added the hard‐masked genes to the corresponding ortholog FASTA file, aligned with MACSE 2.07 (Ranwez et al., 2018), and codons with frameshifts (labeled with “!” by MACSE) were replaced with gaps, and sites that contain only gaps were removed with trimAl 1.2 (‐noallgaps; Capella‐Gutiérrez et al., 2009). We split each ortholog alignment into exons using S*ilene latifolia* hard‐masked genes as a guide, yielding 51,292 exons. We realigned individual exons with OMM_MACSE to verify the coding frame and to clean non‐overlapping ends (minimum 50% coverage), resulting in 28,123 exons. We filtered exon alignments to keep only those of at least 500 bp. We identified and removed sequences that contained a gap proportion greater than 25% of the alignment length using trimAl (get_sequences_gaps_ratio.py), resulting in 3032 exons. We calculated the number of parsimony‐informative sites (PIS) for each exon using PhyKIT 1.15.0 (Steenwyk et al., 2024). We removed exons with PIS less than 20% of total sites, resulting in 2582 exons. To avoid selecting exons from high‐copy gene families, we counted the number of copies of *Moehringia ciliata* and *M. muscosa* in each final homolog tree. We discarded exons from genes with more than two copies of at least one of the species to account for the CARY1 whole‐genome duplication (WGD) in the backbone of Caryophyllaceae (Yang et al., 2018), resulting in a final pool of 2441 exons across Caryophyllaceae. For final exon selection, we retained only exons containing sequences for *Moehringia ciliata* and *M. muscosa*, removing all other sequences. We aligned the resulting 2054 exons with MACSE, codons with frameshifts replaced with gaps, and removed gap‐only sites with the function pxclsq (‐p 1). We imported alignments into Geneious 11.1.5 (Kearse et al., 2012) to manually filter for loci with clear misassemblies, yielding a final set of 830 exons from 780 genes (25 genes had two exons) with pairwise identity between 89% and 99.6% (average of 98.7%) and lengths ranging from 504 to 3801 bp (average of 1228 bp). For bait design, we selected a single *Moehringia* sequence. Bait synthesis (myBaits) was done at Daicel Arbor BioSciences (Ann Arbor, MI, USA). A total of 18,485 biotinylated 100‐nucleotide RNA probes were designed with a 2× tiling density to capture 1,018,167 bp.

### Taxon sampling

For target enrichment data generation, we included 65 accessions representing all 15 species of *Moehringia* sect. *Moehringia* (including two subspecies of *M. bavarica*). The geographical origin of these samples is shown in Appendix S2: Figure S1. We also included 23 accessions representing seven species of *Moehringia* sect. *Latifoliae*. Additionally, we included two samples of *Arenaria* and one of *Cerastium* (Appendix S1: Table S2). For phylogenomic analyses, we also included the 22 species of Caryophyllaceae and three outgroups used for nuclear tree‐based orthology in target enrichment bait design (Appendix S1: Table S1).

### DNA extraction, library preparation, target enrichment, and sequencing

We extracted total genomic DNA using the Qiagen DNeasy Plant Mini Kit (Qiagen, Venlo, Netherlands) and the manufacturer's protocol. We performed no RNase treatment and performed the final elution twice, each time using 25 µL of 10 mM Tris‐HCl. We quantified genomic DNA using a Qubit 4 Fluorometer with a dsDNA HS Assay Kit (Thermo Fisher Scientific, Waltham, MA, USA). We qualified genomic DNA by gel electrophoresis on a 0.8% agarose gel in TAE for 30 min at 120 V, with 20–50 ng of DNA sample. For samples with low degradation, we sheared DNA to a fragment size of 350 bp using a Covaris M220 Focused‐ultrasonicator (Covaris, Woburn, MA, USA). We performed library preparation using the NEBNext Ultra II DNA Library Prep Kit for Illumina (New England Biolabs, Ipswich, MA, USA) and the NEBNext Multiplex Oligos for Illumina (Index Primers Set 2; New England Biolabs) according to the manufacturer's protocol but using only half the recommended volumes to reduce the cost per sample. We amplified adaptor‐ligated libraries with 8–10 PCR cycles. We treated all libraries derived from highly degraded DNA with an alternative cleanup using 0.9× the bead volume. We quantified libraries using the Qubit, and quantified fragment distributions on an Agilent 4150 TapeStation System with HSD1000 ScreenTapes (Agilent Technologies, Santa Clara, CA, USA). Before hybridization, we pooled libraries to include 0.325–3.2 µg of total DNA, with 10–16 libraries per pooled library. We concentrated the library pools using a SpeedVac centrifuge until all liquid had evaporated, then re‐eluted them with 7 µL of 10 mM Tris‐HCl. We performed solution‐based hybridization and enrichment in accordance with the MyBaits Hybridization Capture for Targeted NGS User Manual, version 5.02. We used a hybridization temperature (TH) of 60°C and an incubation time of 16 h. We performed the bait‐target hybridized pool bead clean‐up at a wash temperature (TW) of 60°C. Subsequently, we amplified the hybridized libraries for 10–14 PCR cycles with an annealing temperature (TA) of 60°C. We cleaned amplified hybridization reactions with 1× Agencourt AMPure XP beads and two 70% v/v ethanol washes. We quantified the final enriched pooled library concentration and fragment size distribution using the Qubit and TapeStation, respectively. We combined the final libraries into a single pool. Novogene UK (Cambridge, UK) performed sequencing on the Illumina NovaSeq X Plus platform with 150‐bp paired‐end reads, yielding 300 Gb of raw data.

### Nuclear read processing and loci assembly

We assessed the quality of target enrichment raw reads using FastQC 0.12.1 (Andrews, 2010) and MultiQC 1.23 (Ewels et al., 2016). Then we removed PCR duplicates using ParDRe 2.2.5 (González‐Domínguez and Schmidt, 2016) with default parameters. We used CAPTUS 1.0.1 (Ortiz et al., 2026) to trim sequencing adaptors and low‐quality bases, assemble contigs, and extract nuclear loci. We trimmed the leading and trailing read regions with an average PHRED quality score below 20 (‐‐trimq) using BBDuck from BBTools 39.06 (Bushnell et al., 2017). For the assembly step, we used a minimum contig depth (‐‐min_count) of 20 in MEAGHIT 1.2.9 (Li et al., 2015). For the extraction of the 830 nuclear exons, we set the minimum percentage of identity (‐‐nuc_min_identity) in Scipio 1.4.1 (Keller et al., 2008) to 85, 80, and 70 for *Moehringia* sect. *Moehringia*, *Moehringia* sect. *Latifoliae*, and *Arenaria* + *Cerastium*, respectively. Additionally, we set the minimum coverage (‐‐nuc_min_coverage) to 70, the minimum Scipio score (‐‐nuc_min_score) to 30, and the paralog tolerance for retaining gene copies (‐‐paralog_tolerance) to 5 for all locus extractions. Finally, we generated CDS FASTA files for each locus, while limiting each sample to a maximum of five paralogs (‐m NUC ‐f NT ‐‐max_paralogs 5 ‐‐collect_only).

### Orthology inference, phylogenetic reconstruction of nuclear loci, and assessment of discordance and hybridization

For phylogenetic analyses, we merged the corresponding sequences for each of the 830 exons from the target enrichment‐assembled data set and from the Caryophyllaceae transcriptome and genome tree‐based orthologs to obtain a combined data set of 116 samples (113 Caryophyllaceae and three outgroups; Appendix S1: Tables S1 and S2). We aligned individual loci with MACSE and replaced codons with frameshifts (labeled with “!” by MACSE) with gaps. Additionally, we cleaned aligned columns to have no more than 90% missing data using phyx. We inferred homolog trees using IQ‐TREE 2.3.6 (Minh et al., 2020) with an extended model selection (Kalyaanamoorthy et al., 2017) and no clade support. We performed monophyletic and paraphyletic masking, outlier detection and removal, and orthology inference in the same way as for the nuclear tree‐based orthology used in target enrichment bait design. We retained only orthologs with at least 39 (one‐third of ingroup samples) samples present. We aligned individual ortholog loci with MACSE, cleaned with phyx, and inferred trees with IQ‐TREE using extended model selection and 1000 ultrafast bootstrap (UFBoot; Hoang et al., 2018) replicates for node support. We collapsed nodes from ortholog gene trees with a UFBoot support less than 50% and used them to infer a coalescent‐based species tree with ASTRAL‐IV 1.19.4.5 (Zhang et al., 2025) using local posterior probabilities (LPP; Sayyari and Mirarab, 2016) to assess clade support and an average gene sequence length of 1000 for branch length estimation in substitution units with CASTLES‐2 (Tabatabaee et al., 2023; integrated with ASTRAL‐IV).

We visualized gene tree discordance by first calculating the number of concordant and discordant bipartitions on each node of the ASTRAL‐IV species tree using Phyparts (Smith et al., 2015). We used individual gene trees with UFBoot support of at least 70% for each node. We plotted pie charts, taking into consideration uninformative and missing nodes, using the script phypartspiecharts_missing_uninformative.py (Morales‐Briones et al., 2022). To distinguish conflict from poorly supported branches, we carried out a quartet sampling (QS; Pease et al., 2018) analysis using a concatenated matrix of all loci, a partition‐by‐loci (‐‐genetrees) scheme, the ASTRAL‐IV species tree, 5000 replicates, and RAxML‐NG 1.2.2 as the ML engine (Kozlov et al., 2019). The QS evaluates the relative support among the three possible quartet topologies for a node (quartet concordance, QC), the disparity between the sampled proportions of the two discordant quartet topologies (quartet differential, QD), and quantifies the proportion of informative quartet replicates (quartet informativeness, QI).

To explore common sources of gene tree discordance (incomplete lineage sorting [ILS] and introgression/hybridization [IH]), we used Phytop 0.3 to estimate ILS and IH indices. Phytop uses ASTRAL's local quartet frequencies around each internal branch and relies on the expectation that under ILS the two discordant quartet topologies occur at similar frequencies (q2 ≈ q3). In contrast, introgression/hybridization can generate asymmetric discordance (q2 ≠ q3). Phytop partitions discordance into ILS and IH components based on whether q2 and q3 are statistically distinguishable. We inferred species trees with ASTRAL‐IV as described above, adding the option ‐u 2 to output local quartet frequencies (q1, q2, q3) for each internal branch. We used this annotated ASTRAL‐IV tree as input to Phytop with all parameters set to their default values.

Finally, to test for hybridization directly from gene trees, we used the recently described phylogenetic network method CAMUS (Constrained Algorithm Maximizing qUartetS; Willson and Warnow, 2026). CAMUS overcomes the scalability limitations of many commonly used phylogenetic network methods, which are often restricted to relatively small data sets, by using an input constraint tree (e.g., a coalescent‐based species tree) and a set of unrooted quartets derived from the gene trees. CAMUS then estimates a rooted phylogenetic network built on the constraint tree by adding reticulation edges to maximize the number of quartet trees satisfied by the network. This framework reduces the need to subsample the data set to test specific hybridization hypotheses. It enables evaluation of networks with increasing numbers of reticulations in a fraction of the time required by many alternative methods. However, CAMUS is limited to level‐1 phylogenetic networks and does not estimate inheritance probabilities. For network estimation, we first pruned the final ortholog gene trees and the ASTRAL‐IV tree to retain only the 65 *Moehringia* sect. *Moehringia* samples from target enrichment plus *Arenaria serpyllifolia* (C35) as an outgroup, resulting in 789 gene trees. We then used CAMUS 1.0.1 with the ASTRAL‐IV tree as the constraint topology and collapsed nodes in the ortholog gene trees with UFBoot ≤0.5. To choose the most suitable number of reticulations, we examined the series of networks returned by CAMUS. We selected the point at which the proportion of satisfied quartets no longer improved substantially as described by Willson and Warnow (2026).

### Divergence time estimation

We estimated divergence times using two approaches. First, we estimated a time‐calibrated tree using the relative rate framework (RRF) RelTime (Tamura et al., 2012) as implemented in MEGA 11.0.13 (Tamura et al., 2021). We calculated confidence intervals (CIs) using the method described by Tao et al. (2020), which is also implemented in RelTime. We used the rooted ASTRAL‐IV tree and the concatenated alignment of all loci as input for this analysis. We set the sequence evolution model to GTR + G with four discrete gamma categories, and the parameter Gaps/Missing Data Treatment to 95%, retaining 75,225 sites from the original alignment for the analysis. We set additional parameters to their default values and set *Beta vulgaris*, *Spinacia oleracea*, and *Phaulothamnus spinescens* as outgroups. We also carried out a dating analysis using a Bayesian approach with an uncorrelated lognormal (UCLN) relaxed clock, as implemented in BEAST 2.7.7 (Bouckaert et al., 2019). We reduced the number of loci to facilitate computational time using SortaDate (Smith et al., 2018). We chose the top 20 loci based on the least topological conflict with the ASTRAL‐IV tree (i.e., bipartition concordance), clock‐likeness (i.e., root‐to‐tip variance), and amount of evolutionary change (i.e., total tree length). The 20‐locus concatenated alignment was 43,254 bp long, with 9% missing data. We set the sequence evolution model to GTR + G with four discrete gamma categories, fixed the topology to the ASTRAL‐IV tree, used a birth–death tree speciation prior, and ran four independent MCMC runs of 200 million generations, sampling every 2000 generations. We assessed parameter estimate convergence across the four independent MCMC runs by analyzing plots of all parameters and ‐lnL after reaching an ESS (effective sample size) ≥200, using Tracer 1.7.2 (Rambaut et al., 2018). We discarded the first 25% of sampled trees as burn‐in. We summarized the trees in TreeAnnotator 2.7.7 to produce a maximum clade credibility chronogram with median divergence time estimates and 95% highest posterior density (HPD) intervals. We calibrated both dating analyses using two fossils. First, we calibrated the crown age of the clade comprising tribes Sileneae, Caryophylleae, Eremogoneae, Alsineae, Arenarieae, Sclerantheae, and Sagineae (corresponding broadly to the former Alsinoideae–Caryophylloideae clade; sensu Harbaugh et al., 2010; Greenberg and Donoghue, 2011) using the inflorescence fossil *Caryophylloflora paleogenica* (Jordan and Macphail, 2003). This fossil, from middle–late Eocene deposits, has morphological features consistent with derived Caryophyllaceae and is therefore interpreted as belonging to crown Caryophyllaceae. However, its precise placement within the family remains uncertain (Jordan and Macphail, 2003). We calibrated this clade using a lognormal prior with an offset of 33.9 Ma, a mean of 0.0, and a standard deviation of 1.0, following Dillenberger and Kadereit (2017). The offset corresponds to the Eocene–Oligocene boundary (33.9 million years ago [Ma]), which we used as a conservative minimum age constraint reflecting the youngest possible age of the fossil‐bearing strata. Additionally, we used the pantoporate pollen fossil *Periporopollenites polyoratus* to constrain the crown age of Caryophyllaceae. This fossil has been reported from Late Cretaceous deposits of the Gippsland Basin, with occurrences assigned to the Campanian (Stover and Partridge, 1973). As done by Schenk et al. (2018) and Feng et al. (2024), we used the Campanian–Maastrichtian boundary (72.1 Ma) as a minimum age constraint. Because *Periporopollenites* is a form taxon with uncertain phylogenetic affinity, this calibration should be interpreted cautiously. We applied a maximum age constraint of 85.7 Myr, based on the 95% highest posterior density estimate of the crown age of Caryophyllaceae from Xue et al. (2023).

### Plastid CDS assembly, tree inference, and haplotype network

We assembled plastid coding sequences (CDS) from off‐target reads of the target enrichment (91 samples; Appendix S1: Table S2) and transcriptome data (18 samples; Appendix S1: Table S1) using CAPTUS and the provided set of plastid proteins (‐p SeedPlantsPTD) as references. Additionally, we used CAPTUS to extract plastid CDS from plastomes available in NCBI GenBank for seven samples (Appendix S1: Table S3). We generated FASTA files for 80 plastid CDS using CAPTUS, retaining only the best hit (‐m PTD ‐f NT ‐‐max_paralogs 0 ‐‐collect_only). We aligned individual plastid CDS using MAFFT 7.526 (‐‐auto; Katoh and Standley, 2013) and removed aligned columns with >90% missing data with phyx (pxclsq). Then, we concatenated all 80 CDS and estimated an ML tree using IQ‐TREE with extended model selection and 1000 UFBoot replicates. Additionally, we used QS with 1000 replicates to assess potential branch conflict. We estimated a plastid haplotype network for the 65 target enrichment accessions representing all 15 species of *Moehringia* sect. *Moehringia* using the “haplotype” function from the R package pegas (Paradis, 2010), with default parameters. As input, we filtered the concatenated plastid alignment with nremover.pl (https://github.com/tkchafin/scripts) to keep only biallelic single nucleotide polymorphisms (SNPs), remove sites with only a singleton SNP, and retain columns with a maximum gap proportion of 5% (‐b ‐s ‐g 0.05). This resulted in a filtered alignment of 25,991 bp with 3% gaps.

### Analyses of population structure and hybridization based on SNPs

We performed SNP calling from the target enrichment data for the 65 accessions of *Moehringia* sect. *Moehringia*. First, we mapped the filtered paired‐end reads from CAPTUS to the chromosome‐scale reference genome of *Moehringia trinervia* (https://portal.darwintreeoflife.org/data/Moehringia%20trinervia) using the algorithm mem of BWA 0.7.18 (Li and Durbin, 2009). We then sorted the resulting BAM files with SAMtools 1.18 (Danecek et al., 2021), merged them with unaligned reads (MergeBamAlignment), marked them for duplicates (MarkDuplicates) with GATK 4.6.0.0 (McKenna et al., 2010), and indexed them with SAMtools. We performed variant calling (HaplotypeCaller) for each sample using GATK in GVCF mode. We merged the resulting GVCF files (CombineGVCFs) and jointly genotyped (GenotypeGVCFs) using GATK. We retained SNPs (SelectVariants) and hard filtered (VariantFiltration – QD < 2.0 || QUAL < 30.0 || SOR > 3.0 || FS > 60.0 || MQ < 40.0 || MQRankSum < –12.5 || ReadPosRankSum < –8.0) with GATK to remove SNPs with low base quality or low coverage. Finally, we used VCFtools 0.1.17 (Danecek et al., 2011) to filter the SNPs further to exclude sites with no alternative allele calling for any of the samples and sites with a proportion of missing data greater than 25%, while keeping only bi‐allelic SNPs and SNPs that are distanced at least 50 bp, with minimum site and genotype quality scores of 30, and masking all genotypes with a depth below 5× and above 150× (‐‐remove‐indels ‐‐max‐missing 0.75 ‐‐minQ 30 ‐‐min‐meanDP 5 ‐‐max‐meanDP 150 ‐‐min‐alleles 2 ‐‐max‐alleles 2 ‐‐thin 50 ‐‐non‐ref‐ac‐any 1).

We identified differentiation axes among all 15 species of *Moehringia* sect. *Moehringia* with principal components analysis (PCA) using PLINK 1.9.0‐b.7.7 (Purcell et al., 2007). We used PLINK with a window size of 5 kb, a window step size of 5 bp, and linkage pruning with an *r* ^2^ threshold of 0.5. Additionally, to obtain PCAs with better distinction among most species, we estimated PCAs after removing the two most distinct species. First, we removed *M. diversifolia* W.D.J. Koch and *M. lebrunii* Merxm., and then further removed *M. argenteria* and *M. sedoides* (Pers.) Loisel. We also used ADMIXTURE 1.3.0 to assess population structure in a maximum likelihood (ML) framework (Alexander et al., 2009). We ran ADMIXTURE using the same input used for the PCA (*K* = 1–16) and performed cross‐validation error estimation (CV = 25) to assess the most suitable value of *K* (Alexander and Lange, 2011).

We explored gene flow among individuals of *Moehringia* sect. *Moehringia* using the site‐pattern‐based *D*‐statistic (ABBA/BABA) and the *f*_4_ admixture ratio (Durand et al., 2011; Patterson et al., 2012) on biallelic SNPs in Dsuite 0.5 (Malinsky et al., 2021). We used the program Dtrios in Dsuite to calculate *D* and *f*_4_ for all possible trios of samples using the ASTRAL‐IV species tree as a guide, and plotted *D* and *f*_4_ with the scripts plot_d.rb and plot_f4ratio.rb, respectively (https://github.com/millanek/tutorials/tree/master/analysis_of_introgression_with_snp_data/). Because Dsuite reports unadjusted trio‐level *P*‐values, we assessed significance at the trio level and controlled the familywise error rate across all tests using the Holm correction (Holm, 1979) in R; adjusted *P*‐values < 0.01 were considered significant. We also used *f*‐branch (Malinsky et al., 2018) to calculate the *f*‐branch statistic and summarize correlated *f*_4_ results, thereby localizing excess allele sharing on specific branches of the phylogenetic tree (Malinsky et al., 2021). We ran Fbranch with the option ‐Z and used the accompanying Zb matrix as an additional measure of branch‐level support; cells with ∣*Z*_*b*_∣ ≥ 2.576 were considered supported at an approximate two‐sided *α* = 0.01. In Dsuite, *Z* denotes the trio‐level *Z*‐score associated with Patterson's *D*‐statistic for each tested sample trio. By contrast, *Z*_*b*_ is the branch‐level *Z*‐score reported by Fbranch and was used here as an additional measure of support for localized *f*‐branch signals on the phylogeny. We plotted *f*‐branch results with Dsuite's dtools.py.

## RESULTS

### Target enrichment, nuclear loci assembly, orthology inference, and plastome assembly

We obtained between 5.16 million (*Moehringia tommasinii* Marches. C8) and 36.32 million (*Moehringia ciliata* C41) raw reads, with an average of 12.58 million across the 91 samples used for target enrichment (Appendix S1: Table S4). Overall, we recovered 829 of 830 exons; the loci recovery for *Moehringia* sect. *Moehringia* ranged between 823 (*Moehringia muscosa* C67) and 829 (e.g., *Moehringia muscosa* C10), with an average of 828.37, between 817 (*Moehringia trinervia* C36) and 828 (e.g., *Moehringia pentandra* C68), with an average of 825.30 for sect. *Latifoliae*, and between 733 and 822 for *Arenaria* and *Cerastium* (Appendix S1: Table S4). The number of obtained orthologs was 829 for the full data set, with a mean of 652 orthologs per target enrichment sample and 691.32 for the transcriptome samples. The concatenated nuclear matrix for the full data set comprised 1,073,255 aligned columns, with a character occupancy of 76.82%.

We recovered between 15 (*Moehringia sedoides* C2) and 80 (*Moehringia muscosa* C10, out of 80) plastome CDS, with an average of 62.43, for the 91 samples used for target enrichment (Appendix S1: Table S4). For the transcriptome and reference plastomes, the recovery ranged between 58 (*Moehringia muscosa* trp) and 80 (*Phaulothamnus spinescens* MH286322), with an average of 75.88 (Appendix S1: Table S4). The concatenated plastome CDS matrix for the full data set comprised 68,270 aligned columns, with a character occupancy of 84.62%.

### Nuclear phylogenetic analyses, discordance, hybridization, and divergence time estimation

**Figure 2:**
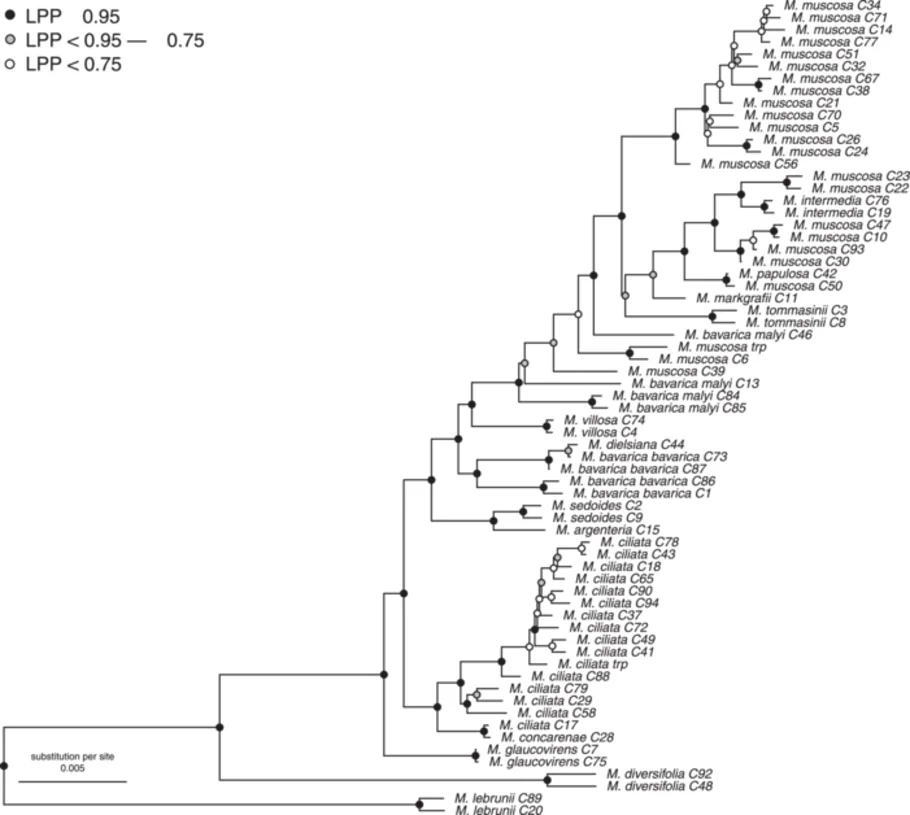
Pruned coalescent‐based species tree of *Moehringia* sect. *Moehringia* inferred with ASTRAL‐IV from 829 orthologous gene trees. Circles on the nodes show local posterior probability (LLP) values. LPP ≥ 0.95 in black; LPP < 0.95 and ≥ 0.75 in grey; LPP < 0.75 in white. Branch lengths in number of substitutions per site (scale bar). See Appendix S2: Figure S2 for the full species tree with all sampled taxa of *Moehringia*, Caryophyllaceae, and outgroups.

The ASTRAL‐IV coalescent‐based species tree showed LPP values above 0.95 throughout the backbone of the tree, but the values were lower for shallow nodes in *Moehringia* sect. *Moehringia* (Figure 2; Appendix S2: Figure S2). Our result showed the monophyly of *Moehringia* and its two sections (Appendix S2: Figure S2). Within *Moehringia* sect. *Moehringia*, *M. lebrunii* was placed as the sister to the rest of the section, followed by *M. diversifolia* and *M. glaucovirens* Bertol. as successive sisters to the rest of the section. These relationships were supported by most individual gene trees, as indicated by the Phyparts congruence analyses (Appendix S2: Figure S3). The QS analysis revealed similar patterns, with these nodes receiving support of at least QC ≥ 0.25 and no signal of alternative topologies (i.e., QD ~ 1; Appendix S2: Figure S4). The rest of the tree was divided into two clades, one clade comprising all *M. ciliata* samples, as well as *M. concarenae*, which was sister to a clade comprising all remaining samples. The support for this clade was moderate (QC = 0.1), with no signal of an alternative topology (QD = 0.9), and Phyparts showed most gene trees as incongruent (Appendix S2: Figure S3). The second large clade comprised the remaining species of *Moehringia* sect. *Moehringia*. The QS support for this clade was low (QC = 0.016), but with no apparent signal of an alternative topology (QD = 0.85) and Phyparts showing most gene trees as discordant. Within the clade, most nodes, except for the sister‐group relationship of *M. argenteria* and *M. sedoides*, and several conspecific accessions of *M. bavarica* subsp. *bavarica* and subsp. *malyi*, *M. muscosa*, *M. tommasinii*, and *M. villosa* showed high levels of discordance (i.e., QC ≤ 0.25) and even countersupport (i.e., QC < 0), but only one node, associated with *Moehringia muscosa* (C39), had a signal of an alternative topology (QD = 0.59). In contrast, Phyparts showed a clear signal of discordance from most gene trees. *Moehringia argenteria* and *M. sedoides* formed a sister clade to the remaining species (Figure 2 and Appendix S2: Figures S3 and S4). This clade was followed by successive clades of *M. bavarica* subsp. *bavarica* + *M. dielsiana* Mattf., *M. villosa*, three samples of *M. bavarica* subsp. *malyi* in two successive clades, three samples of *M. muscosa* in two successive clades, and, unexpectedly, one sample of *M. bavarica* subsp. *malyi* (C46). We found no explanation for the placement of this accession of *M. bavarica* subsp. *malyi* within *M. muscosa*. The remaining species formed two subclades. The first subclade comprised *M. tommasinii* and *M. markgrafii* Merx. & Gutermann, which were successively sister to the rest, and seven samples of *M. muscosa*, in which *M. papulosa* Bertol. and *M. intermedia* (Loisel.) Panizzi were nested. The second subclade comprised the remaining 14 *M. muscosa* samples (Figure 2).

The Phytop analysis (Appendix S2: Figure S5) revealed only limited ILS at the MRCA of *Moehringia* sect. *Moehringia* and at the nodes subtending *M. diversifolia* and *M. glaucovirens*. In contrast, discordance at the MRCA of the clade comprising all remaining species was explained primarily by ILS, with only a minor signal of IH. The clade comprising *M. ciliata* and *M. concarenae* showed predominantly ILS‐only signals. However, three nodes also exhibited some IH, with ILS still explaining most of the discordance, particularly at the MRCA of the clade and the subsequent split. The remaining clade showed more nodes with combined ILS and IH signals, concentrated around the splits involving *M. argenteria* + *M. sedoides*, *M. bavarica* subsp. *bavarica* (C1, C86, and C87), *M. villosa*, *M. bavarica* subsp. *malyi* (C46), and the MRCA of the clade containing most *M. muscosa* samples plus *M. intermedia*, *M. papulosa*, *M. markgrafii*, and *M. tommasinii*. In all cases, the ILS signal was stronger than the IH signal.

CAMUS inferred networks with up to 13 reticulations, but the number of satisfied quartets did not improve substantially after four reticulations (Appendix S2: Figure S6). The network with four reticulations (Appendix S2: Figure S7) indicated gene flow between *M. muscosa* (C39) and the clade comprising all *M. ciliata* and *M. concarenae* samples. It also indicated gene flow between *M. tommasinii* and the clade comprising seven samples of *M. muscosa* plus *M. intermedia*, *M. papulosa*, and *M. markgrafii*. The other two reticulation events were located among members of *M. ciliata* and *M. muscosa*, respectively.

**Figure 3:**
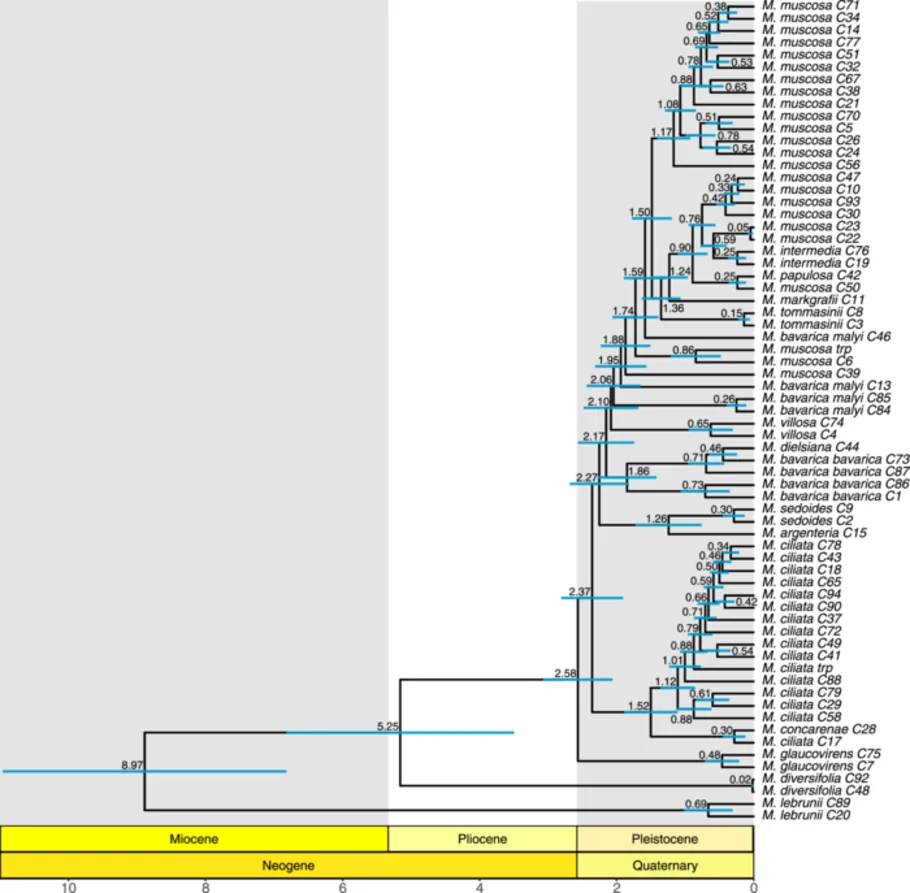
Time‐calibrated pruned tree of *Moehringia* sect. *Moehringia* inferred with BEAST2 based on the ASTRAL‐IV coalescent‐based species tree and a 20‐locus alignment of 43,254 bp. Node labels represent the absolute times of divergence, and blue bars represent the 95% highest posterior density (HPD) intervals. The time unit at the bottom of the geological time scale is in million years. See Appendix S2: Figure S9 for the full time‐calibrated tree with all sampled taxa of *Moehringia*, Caryophyllaceae, and outgroups.

The divergence time estimation conducted with RelTime showed overall younger ages than the BEAST2 analysis, but ages for *Moehringia* sect. *Moehringia* showed a similar pattern regarding the nodes placed in the Miocene, Pliocene, and Quaternary (Appendix S2: Figures S8 and S9). Given recent evidence that RelTime tended to underestimate ages (e.g., Alves et al., 2026), we will focus the following description and discussion on the BEAST2 analyses. Our BEAST2 time‐calibrated phylogeny inferred that *Moehringia* diverged from *Arenaria* at 19.58 Ma (95% HPD: 14.64–25.09; Appendix S2: Figure S9). *Moehringia* sects. *Moehringia* and *Latifoliae* were inferred to have diverged in the Miocene at 11.29 Ma (8.77–14.06; Appendix S2: Figure S9). The crown age of *Moehringia* sect. *Moehringia* (divergence of *M. lebrunii* from the rest of the clade) was inferred to be still in the Miocene at 8.97 Ma (6.55–11.45; Figure 3). The divergence of *M. diversifolia* was inferred to have taken place at 5.25 Ma (3.69–7.01) on the border of the Miocene and Pliocene, and the divergence of *M. glaucovirens* at 2.58 Ma (2.10–3.11) on the border of the Pliocene and Quaternary (Figure 3). All remaining species divergence times were placed in the Quaternary (Figure 3).

### Plastome phylogenetics and haplotype network

The plastome tree (Appendix S2: Figure S10) agreed with the nuclear tree regarding the placement of *M. lebrunii* and *M. diversifolia* in relation to all other species of *Moehringia* sect. *Moehringia*. This placement was highly supported by LPP values and QS scores (Appendix S2: Figures S10 and S11). The remaining species were divided into two clades, which are again strongly supported by LPP and QS. Unlike the nuclear tree (Figure 2), species relationships within these clades did not show any clear pattern, and samples of the same species were sometimes placed in widely separated clades. Furthermore, LPP values and QS scores were generally low within the two clades (Appendix S2: Figures S10 and S11).

**Figure 4:**
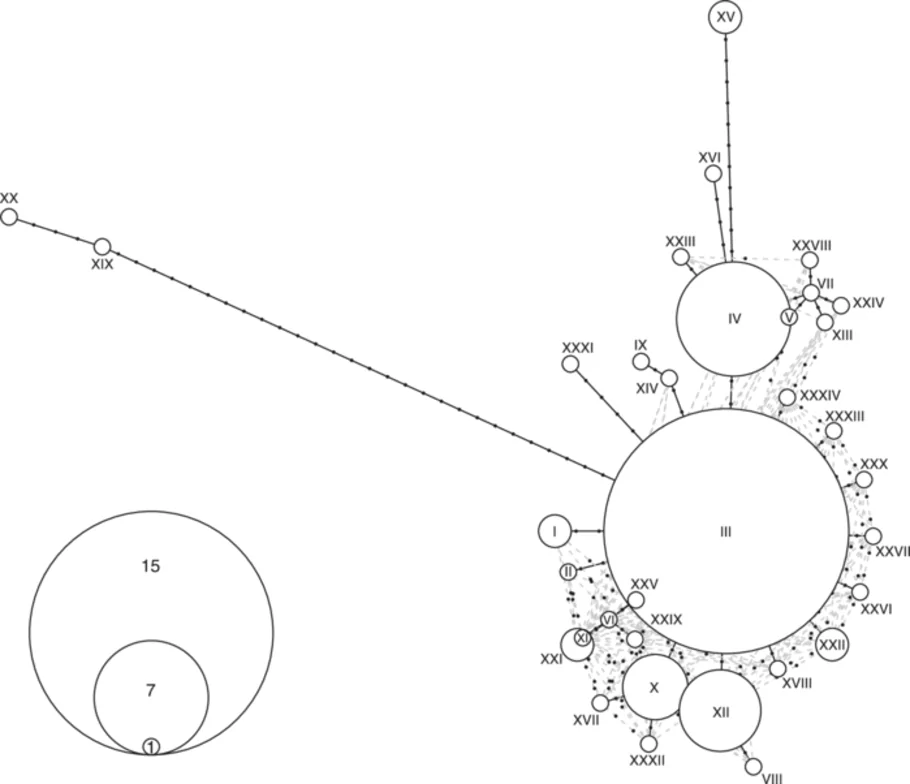
Plastid haplotype network of the 65 target‐enrichment accessions of *Moehringia* sect. *Moehringia*. Each circle represents one haplotype (34 in total), with circle size proportional to haplotype frequency (scale at bottom left). Solid black lines represent the backbone links of the haplotype network. Gray dashed lines indicate alternative plausible links among haplotypes. Black dots along branches represent inferred mutational steps between haplotypes. See Appendix S1: Table S5 for the distribution of haplotypes among taxa.

The 65 target enrichment samples from *Moehringia* sect. *Moehringia* were clustered in 34 haplotypes (Figure 4, Appendix S1: Table S5). Apart from haplotype XV, found in *M. diversifolia*, and haplotypes XIX and XX, found in *M. lebrunii*, only a few mutational steps separated most haplotypes. The most common haplotype III was found in samples of *M. bavarica* subsp. *bavarica*, *M. bavarica* subsp. *malyi*, *M. ciliata*, *M. intermedia*, and *M. muscosa*, and haplotypes I (*M. argenteria*, *M. sedoides*), II (*M. bavarica* subsp. *bavarica*), VI (*M. bavarica* subsp. *bavarica*), VIII (*M. bavarica* subsp. *bavarica*), X (*M. ciliata*, *M. markgrafii*, *M. muscosa*), XI (*M. ciliata*), XII (*M. ciliata*, *M. muscosa*, *M. tommasinii*), XIV (*M. concarenae*), XVII (*M. glaucovirens*), XVIII (*M. intermedia*), XX (*M. lebrunii*), XXI (*M. muscosa*), XXII (*M. muscosa*), XXV (*M. muscosa*), XXVI (*M. muscosa*), XXVII (*M. muscosa*), XXIX (*M. muscosa*), XXX (*M. papulosa*), XXXI (*M. sedoides*), XXXII (*M. tommasinii*), XXXIII (*M. villosa*), and XXXIV (*M. villosa*) were directly or indirectly derived from haplotype III. Haplotype IV was found in samples of *M. bavarica* subsp. *malyi*, *M. ciliata*, *M. dielsiana*, and *M. muscosa*, and haplotypes V (*M. bavarica* subsp. *bavarica*), VII (*M. bavarica* subsp. *bavarica*), XIII (*M. ciliata*), XVI (*M. glaucovirens*), XXIII (*M. muscosa*), XXIV (*M. muscosa*), and XXVIII (*M. muscosa*) were directly or indirectly derived from haplotype IV.

### SNP analyses of population structure and hybridization

**Figure 5:**
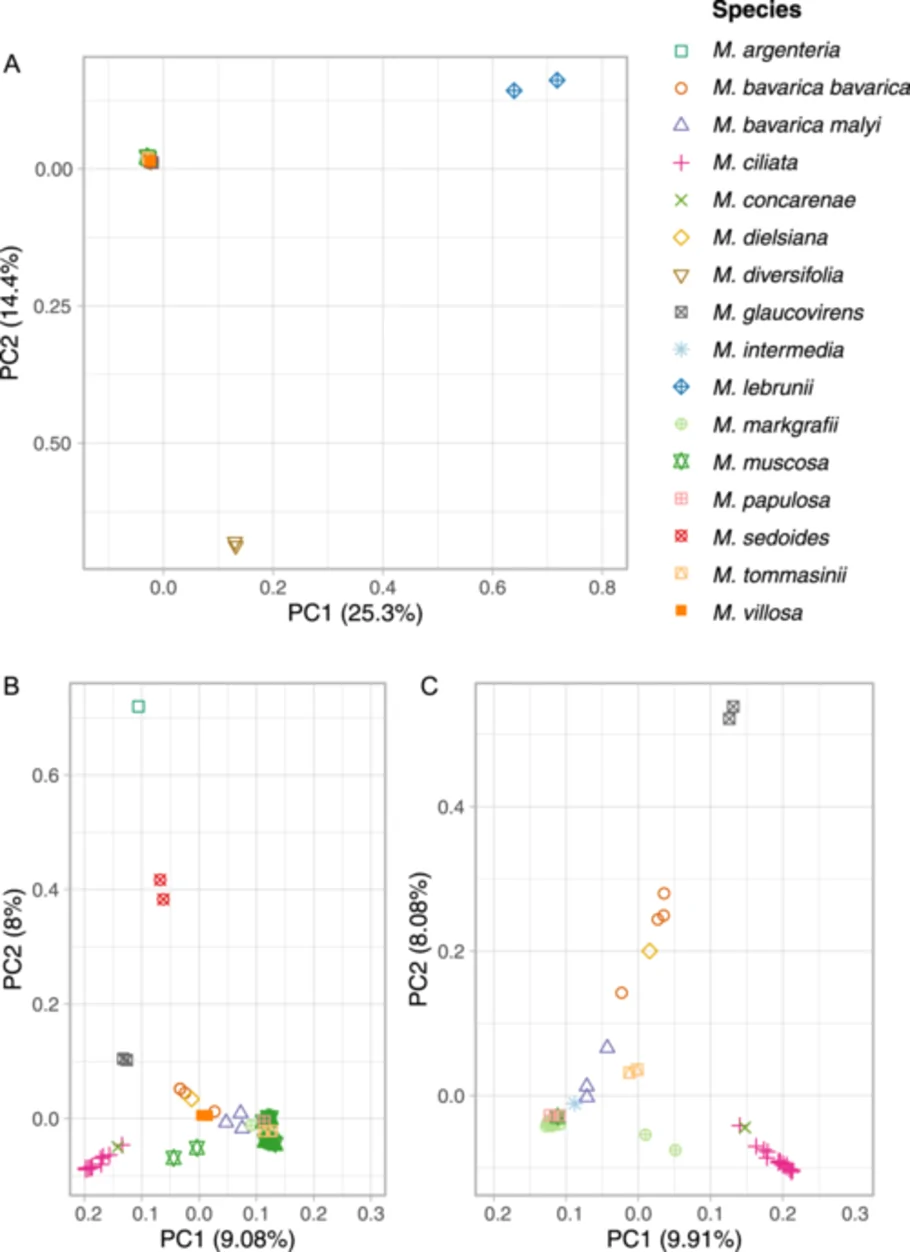
(A) PCA of all species (65 target enrichment accessions) of *Moehringia* sect. *Moehringia* based on 44,304 genome‐wide SNPs inferred with PLINK, showing the two main axes of differentiation. (B) PCA after removal of *M. diversifolia* and *M. lebrunii*, based on 35,825 SNPs. (C) PCA after further removal of *M. argenteria* and *M. sedoides* based on 33,468 SNPs.

The PCA of genome‐wide SNP data of all 65 target enrichment samples of *Moehringia* sect. *Moehringia* (44,304 SNPs; Figure 5A) showed a clear split between *M. lebrunii* and *M. diversifolia* and the rest of the species. Most of the differentiation was shown by PC1 and PC2 (Figure 5; Appendix S2: Figure S12), and the remaining species of the section were clustered very tightly. Upon removal of *M. lebrunii* and *M. diversifolia* (61 samples and 35,825 SNPs; Figure 5B), all PC axes showed less differentiation (Figure 5; Appendix S2: Figure S12), but *M. argenteria* and *M. sedoides* were clearly separated from the other species. Removal of these last two species (58 samples and 33,468 SNPs; Figure 5C) resulted in a better distinction between most species, i.e., *M. bavarica* subsp. *bavarica*, *M. bavarica* subsp. *malyi*, *M. glaucovirens*, *M. ciliata*, *M. concarenae*, *M. dielsiana*, *M. markgrafii*, and *M. villosa*. All samples of *M. muscosa* (except C6 and C39) formed a tight cluster with *M. intermedia*, *M. papulosa*, and *M. tommasinii*.

Cross‐validation error estimates indicated that *K* = 9 was the optimal number of populations (Appendix S2: Figure S13). The ADMIXTURE analyses based on 44,304 genome‐wide SNPs are shown in Appendix S2: Figure S14. Although several individuals showed mixed ancestry, those species nested within *M. muscosa* and *M. ciliata* in the phylogenetic tree at *K* = 9 showed either no admixture (*M. concarenae*, *M. intermedia*, *M. papulosa*, *M. tommasinii*) or no admixture with species other than *M. muscosa* (*M. markgrafii*).

**Figure 6:**
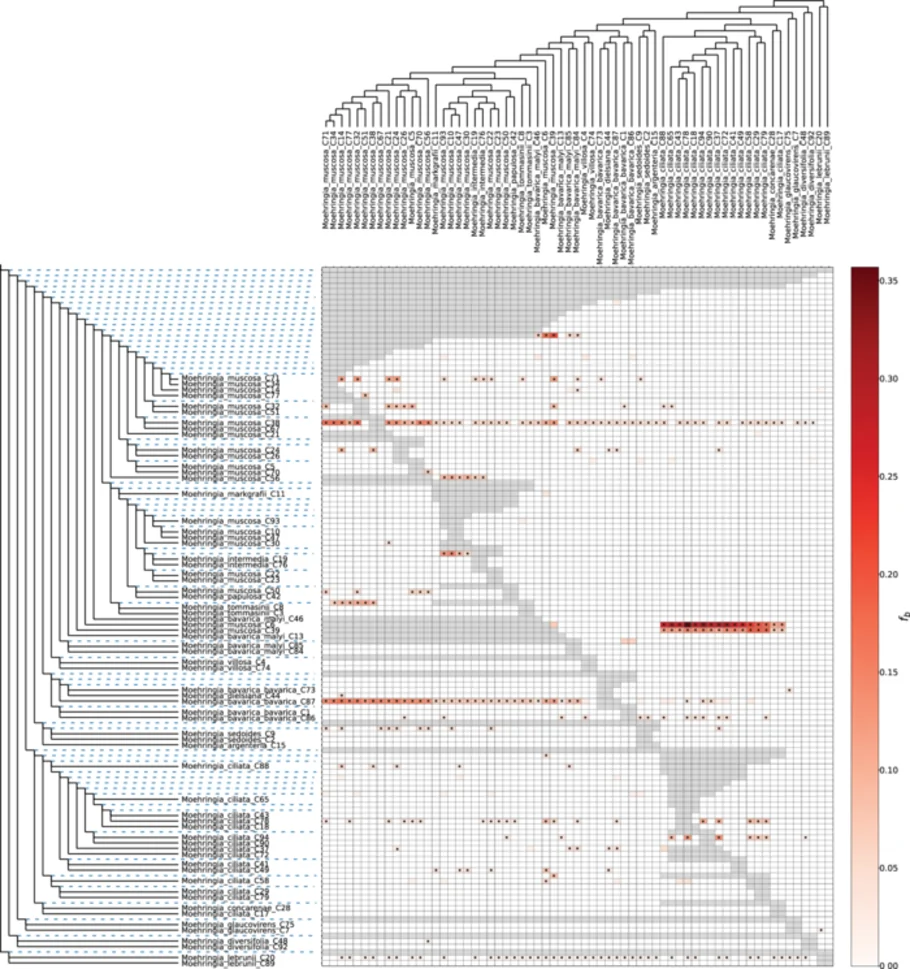
The *f‐*branch statistics for members of *Moehringia* sect. *Moehringia*. The species tree used is shown along the *x*‐ and *y*‐axes. Dotted blue lines in the tree on the *y*‐axis represent ancestral lineages. Darker colors depict increasing evidence for gene flow between lineages. Grey squares represent trio combinations incompatible with the species tree. Significant branch‐level *Z*_*b*_ signals are indicated with a black dot (⋅) in the corresponding cell (see also Appendix S1: Table S7).

The *D*‐statistic, *f*_4_ admixture ratio, and *f*‐branch analyses revealed a few instances of gene flow among members of sect. *Moehringia* (Figure 6; Appendix S1: Table S6; Appendix S2: Figure S15). After Holm correction, significant trio‐level tests supported a clear instance of gene flow involving samples C6 and C39 of *M. muscosa* and all members of *M. ciliata* plus *M. concarenae*, which together explained the phylogenetic position and population structure of those two samples relative to the remaining members of *M. muscosa*. They agreed with the reticulation event detected with CAMUS. Also, the ancestral branch of *M. tommasinii* showed gene flow with six samples of *M. muscosa*, which are not the same as those involved in the reticulation inferred by CAMUS. Additional gene flow was inferred between sample C87 of *M. bavarica* subsp. *bavarica* and mainly with a clade of 14 individuals of *M. muscosa*. The corresponding *f*‐branch/Zb results are provided in Appendix S1: Table S7 as branch‐level support for these localized excess allele‐sharing signals.

## DISCUSSION

Time of diversification and geographical distribution of endemics apart from the divergence of *M. lebrunii* from the Maritime Alps, dated to (6.55–)8.97(–11.45) Ma, and of *M. diversifolia* from the easternmost Central Alps, dated to (3.69–)5.25(–7.01) Ma, the diversification of *Moehringia* sect. *Moehringia* clearly took place at the boundary of the Pliocene and Quaternary and began at (1.94–)2.37(–2.84) Ma (Figure 3). As found here for *M. lebrunii* and *M. diversifolia*, other endemics from the Alps are of pre‐Quaternary age (Patsiou et al., 2014; de Queiroz et al., 2022; Kadereit, 2024), and other notable and largely Quaternary radiations in the Alps have been shown to have started before the onset of the Quaternary (*Androsace*: Schneeweiss et al., 2004; Boucher et al., 2016b; *Facchinia*: Dillenberger and Kadereit, 2017; *Phyteuma*: *Primula* L. sect. *Auricula*: Boucher et al., 2016a, b, but see Zhang et al., 2004; *Saxifraga* subsect. *Arachnoideae*: Gerschwitz‐Eidt et al., 2023).

Most regionally or locally distributed species of *Moehringia* sect. *Moehringia* are found in known refugial areas. Thus, *M. intermedia* grows in eastern Provence; *M. lebrunii*, *M. sedoides*, and *M. argenteria* in the Maritime Alps; and *M. tommasinii* in Istria, all areas identified as refugial by Médail and Diadema (2009). *Moehringia bavarica* subsp. *bavarica* (sensu Kadereit, 2025) grows in the Giudicarian Alps and the Bergamo Alps; *M. concarenae* and *M. dielsiana* in the Bergamo Alps; *M. glaucovirens* with a disjunct range in the Carnic Alps/Dolomites and the Giudicarian Alps; *M. markgrafii* in the Giudicarian Alps, *M. diversifolia* in the easternmost Central Alps, and *M. villosa* in the Julian Alps. All these areas are refugia according to Tribsch and Schönswetter (2003). Although the range of *M. papulosa* in the central Apennine is not known for a concentration of endemics, the range of the species in this area is peripheral to that of the widespread *M. muscosa*. The distribution of at least some Quaternary‐age species in well‐known peripheral glacial refugia is here taken as evidence for the role of Quaternary climatic oscillations in their evolution, assuming that these areas served as refugia in Quaternary glacials and have been inhabited since then (Schönswetter et al., 2005). Indeed, modeling of distribution ranges during the Last Glacial Maximum (LGM) in other plant lineages has shown that their LGM ranges were geographically close to or included their current refugial ranges (Schorr et al., 2012, 2013; Casazza et al., 2013; Patsiou et al., 2014; Pan et al., 2020; Guerrina et al., 2022; Adamo et al., 2023).

Interestingly, species of different ages are distributed in the same refugial area, as is most evident for *M. lebrunii* (8.97 Ma) and *M. sedoides*/*M. argenteria* (2.27 Ma) in the Maritime Alps, but also for *M. glaucovirens* (2.58 Ma) and *M. markgrafii* (1.24 Ma) in the Alpi Giudicarie. This pattern of species of different ages occupying the same refugial areas probably indicates that certain areas served as refugia at different times during the Quaternary.

### The geographical setting of the origin of endemics is derived from within other species

Of the refugial endemics, the tetramerous *M. intermedia*, *M. markgrafii*, *M. papulosa*, and *M. tommasinii* are nested in the tetramerous and widespread *M. muscosa* and originated at 0.69, 1.24, 0.25, and 1.36 Ma, respectively. More specifically, all four species are nested within (*M. intermedia*, *M. papulosa*) or are successive sisters to (*M. tommasinii*, *M. markgrafii*) a group of *M. muscosa* accessions (C10, C22, C23, C30, C47, C50, C93), which originated from areas west of Turin and the Apennine (Appendix S2: Figure S1). *Moehringia papulosa* contains a plastid haplotype (XXX; Appendix S1: Table S5) otherwise unknown in the material we studied. *Moehringia intermedia* contains one unique haplotype (XVIII) and one haplotype (III) also known from *M. ciliata*, *M. muscosa*, and *M. bavarica*, and *M. tommasinii* again contains one unique haplotype (XXXII) and one haplotype (XII) also known from *M. ciliata* and *M. muscosa*. The haplotype of *M. dielsiana* (X) is also known from *M. ciliata* and *M. muscosa*. The presence of unique haplotypes in three of the four species nested within *M. muscosa* provides additional evidence of their evolutionary distinctiveness.

Considering that *M. intermedia*, *M. markgrafii*, *M. papulosa*, and *M. tommasinii* are essentially distributed within (or adjacent in the case of *M. papulosa*) the range of *M. muscosa*, which additionally is likely to have shown range changes in Quaternary times, their phylogenetic position within *M. muscosa* may also be the result of hybridization. This possibility is not supported by our ADMIXTURE and gene flow analyses (Appendix S2: Figures S14 and S15), which showed that, among these four species, three (*M. intermedia*, *M. papulosa*, *M. tommasinii*) showed no evidence of hybridization. Although the fourth species (*M. markgrafii*) showed admixture, this involved only *M. muscosa*. Also, *M. intermedia*, *M. papulosa*, and *M. tommasinii* are not separated from *M. muscosa* in our PCA, in which, however, *M. markgrafii* occupies a position somewhat between *M. muscosa* and *M. bavarica* ssp. *malyi*. In the CAMUS analysis (Appendix S2: Figure S7), gene flow between *M. tommasinii* and the clade comprising seven samples of *M. muscosa* plus *M. intermedia*, *M. papulosa*, and *M. markgrafii* was inferred. In addition, hybridization in *Moehringia* sect. *Moehringia* has only been described between *M. ciliata*, *M. muscosa*, and *M. bavarica* subsp. *bavarica* (Hind, 1988), and hybrids between *M. muscosa* and *M. tommasinii* have recently been reported by Surina et al. (2023). However, the *M. tommasinii* material we sampled originated from populations not affected by hybridization with *M. muscosa* (Surina et al., 2023). In conclusion, we can confidently exclude hybridization as the cause of the phylogenetic placement of *M. intermedia*, *M. markgrafii*, and *M. papulosa* within *M. muscosa*. Evidence for *M. tommasinii* is less clear for two reasons. First, as discussed above, gene flow between *M. tommasinii* and a subclade of *M. muscosa* was detected in the CAMUS analysis. Second, this analysis detected gene flow between *M. muscosa* C39 and the ancestor of *M. ciliata* and *M. concarenae*, and admixture between *M. muscosa* C6 and C39 in the ADMIXTURE analysis (Appendix S2: Figure S14). This admixture may be the cause of the positions of *M. muscosa* C6 and C36, which contribute to the placement of *M. tommasinii* within *M. muscosa*. Of the pentamerous species, *M. concarenae* originated from within the widespread *M. ciliata* at 0.30 Ma, and *M. dielsiana* originated from within the regionally distributed *M. bavarica* subsp. *bavarica* at 0.46 Ma. Our ADMIXTURE and gene flow results provide no evidence of hybridization in *M. dielsiana*, and the admixture observed in *M. concarenae* involved only clusters also present in *M. ciliata* (Appendix S2: Figure S14).

Because it is well established that peripatric (or “budding”) speciation can result in paraphyly of the progenitor species (Rieseberg and Brouillet, 1994; Funk and Omland, 2003; Rosenberg, 2003; Mallet, 2007; Freudenstein et al., 2017), and considering the geographical distribution and phylogenetic relationships of *M. intermedia*, *M. markgrafii*, *M. papulosa*, and *M. tommasinii*, these species most likely originated by peripatric speciation from *M. muscosa*, which as the progenitor of these four species is still extant. The same considerations apply to the origin of *M. concarenae* and *M. dielsiana*. The time from paraphyly to monophyly of such progenitor species depends strongly on population size (Rosenberg, 2003), with smaller populations reaching monophyly faster than larger ones. Given the habitats and distributions of *M. ciliata* and *M. muscosa*, these species may have very large population sizes. Because the progenitor species identified here (*M. muscosa*, *M. ciliata*, *M. bavarica* subsp. *bavarica*) have not formed a monophyletic group, it seems justified to refer to the pattern as incomplete speciation. Denser sampling, particularly of *M. muscosa* and *M. bavarica* subsp. *bavarica* might have revealed those areas within the range of these two taxa from where the derived endemics might have originated.

In the Alps, a pattern very similar to what we found for *M. muscosa* had been identified in *Heliosperma pusillum* (Waldst. & Kit.). Rchb., for which it was shown that montane ecotypes (sometimes classified as a different species) originated up to five times independently from alpine populations (Trucchi et al., 2017). This transition was dated to the Holocene. In *Alyssum* L. from the Alps, the (sub)montane *A. wulfenianum* Willd. subsp. *wulfenianum* was shown to have originated from within the (sub)alpine *A. wulfenianum* subsp. *ovirense* (A.Kern.) Magauer, Schönswetter & Frajman in adaptation to warmer, drier habitats (Magauer et al., 2014).

### Ecological and morphological divergence of endemics derived from within other species

In the case of *M. muscosa* and its four derivative species, there exists strong habitat divergence. Whereas *M. muscosa*, growing at subalpine to alpine elevations (Hind, 1988) and reaching elevations up to 2300 m a.s.l. (Friedrich, 1969), is a species growing in damp calcareous soils frequently found in heavily shaded areas among damp mossy rocks or on woodland floors or alongside small streams among moss (Hind, 1988), and occasionally also grows in crevices or under overhanging rocks on damp and shady vertical cliffs (Friedrich, 1969), *M. intermedia* (650 m a.s.l.), *M. markgrafii* (305 m), *M. papulosa* (200 m) and *M. tommasinii* (100–350 m) are mostly found in crevices of vertical or overhanging calcareous cliffs (Friedrich, 1969; Hind, 1988) which, as far as known (Martini, 1990; Biondi and Bianchelli, 2008; Landolt et al., 2010; Soriano et al., 2012; Fišer et al., 2014; Table 1), are drier than those of *M. muscosa* and located at rather low elevations. While *M. muscosa* is not succulent, *M. intermedia*, *M. markgrafii*, *M. papulosa*, and *M. tommasinii* all are succulent to a varying degree, and *M. papulosa* and *M. tommasinii* also are glaucous, as occasionally is *M. intermedia*. However, succulence is somewhat plastic in sect. *Moehringia*. Thus, Merxmüller and Grau (1967), based on cultivation experiments of species of sect. *Moehringia*, concluded that not succulence itself, but the degree of succulence in optimal growing conditions, is species‐specific. It seems plausible to interpret the succulence and glaucousness of these four derivative species as adaptations to a drier habitat.

Of the pentamerous endemics derived from within other species, *M. concarenae* is ecologically little differentiated from *M. ciliata*, and both grow on calcareous scree (Friedrich, 1969; Hind, 1988; Fenaroli and Martini, 1992) at subalpine to alpine elevations. However, Landolt et al. (2010) assessed the habitat of *M. concarenae* as drier than that of *M. ciliata*. The distributions of *M. concarenae* and *M. ciliata* are non‐overlapping (Fenaroli and Martini, 1992). The distinction between *M. ciliata* and *M. concarenae* is difficult and based mostly on quantitative characters. However, the two species appear to differ in genome size (P. Schönswetter, University of Innsbruck, personal communication). *Moehringia dielsiana* appears to inhabit the same habitat as *M. bavarica* subsp. *bavarica*. Again, the distribution of *M. dielsiana* is non‐overlapping with the distribution of *M. bavarica* subsp. *bavarica* (Mattfeld, 1925; Friedrich, 1969).

It thus seems possible that the divergence of *M. intermedia*, *M. markgrafii*, *M. papulosa* and possibly *M. tommasinii* from *M. muscosa* and the divergence of *M. concarenae* from *M. ciliata*, has an adaptive component. For the first four species, their habitat may have provided shelter from adverse conditions during glacial periods. However, better data and population‐level sampling, which enable the detection of natural selection, are needed to support this hypothesis.

### Endemics not derived from other species

All endemics not derived from a widespread species, i.e., *M. lebrunii*, *M. diversifolia*, *M. glaucovirens*, *M. sedoides*, *M. argenteria*, *M. bavarica* subsp*. bavarica*, and *M. villosa*, which are all limited to known refugial areas as already pointed out above, mostly inhabit habitats similar to those of *M. intermedia*, *M. markgrafii*, *M. papulosa*, and *M. tommasinii* described above, i.e., crevices in vertical to overhanging cliffs. In terms of bedrock preference, only *M. diversifolia* and *M. argenteria* grow on siliceous rocks, whereas all others grow on calcareous rocks. Again, similar to those species nested in *M. muscosa*, *M. lebrunii*, *M. glaucovirens*, *M. sedoides*, *M. argenteria*, *M. bavarica* subsp. *bavarica*, *M. dielsiana*, and *M. villosa* are somewhat to distinctly succulent (but see Merxmüller and Grau, 1967 for *M. glaucovirens*), and *M. lebrunii*, *M. glaucovirens*, *M. sedoides*, *M. argenteria*, and *M. bavarica* subsp. *bavarica* and *M. dielsiana* are also glaucous (Merxmüller and Grau, 1967; Friedrich, 1969; Hind, 1988; Table 1).

The habitat and morphology of these endemics can be interpreted in two ways. First, it is conceivable that these species originated in much the same way as hypothesized for *M. intermedia*, *M. markgrafii*, *M. papulosa*, and *M. tommasinii*, i.e., by peripatric speciation from a widespread ancestor. In contrast to the situation in *M. muscosa*, which, as the progenitor of refugial endemics, has not yet reached monophyly, all species discussed here are older, leaving their respective ancestor enough time to coalesce to monophyly. Also, the ancestor may simply have gone extinct. Considering the (comparatively) great age, particularly of *M. lebrunii* and *M. diversifolia*, the current distribution of all these species in known refugia need not mean that they originated in these areas. Second, while the ecology of *M. intermedia*, *M. markgrafii*, *M. papulosa*, and *M. tommasinii* clearly is derived in comparison to that of the ancestral *M. muscosa*, it seems possible that the very similar ecology (and associated morphology) of *M. lebrunii*, *M. diversifolia*, *M. glaucovirens*, *M. sedoides*, *M. argenteria*, *M. bavarica* subsp. *bavarica*, and *M. villosa* is ancestral in sect. *Moehringia*. This hypothesis finds some support in the observation that species of *Moehringia* sect. *Latifoliae*, the sister clade of sect. *Moehringia*, such as *M. pendula* (Waldst. & Kit.) Fenzl and *M. grisebachii* Janka/*M. jankae* Griseb. ex Janka are sister to the rest of the species in sect. *Latifoliae* and grow in rocky habitats, with *M. pendula* mostly found in rock crevices. However, of these three species, only *M. grisebachii* has been described as slightly succulent (Hind, 1988, 1993). Additionally, lineages that are sister to the rest of the species within *Arenaria* L. s.s. (Sadeghian et al., 2015), which in turn is sister to *Moehringia* (Fior et al., 2006; Fior and Karis, 2007; Harbaugh et al., 2010; Greenberg and Donoghue, 2011; Dillenberger and Kadereit, 2014), primarily grow in dry habitats, mainly in southwestern Europe. Beyond this rather general characterization of habitat, we found coding of habitat, elevational distribution, and morphological characters to be difficult because most of these characters are quantitative and strongly overlapping. Accordingly, we did not attempt to reconstruct ancestral states in *Moehringia* sect. *Moehringia*. If this second of our interpretations should be correct, i.e., if crevices on cliffs should be the ancestral habitat of sect. *Moehringia*, the species of very similar ecology and morphology (succulence, partly glaucousness) distributed in refugial areas mostly along the southern edge of the Alps, would result from very different evolutionary histories. While the ecology and morphology of species such as *M. lebrunii* would be ancestral, the very same ecology and morphology of species like *M. intermedia*, *M. markgrafii*, *M. papulosa*, and *M. tommasinii* would be clearly derived and, given their origin within *M. muscosa*, represent a reversal. However, in both interpretations, the same ecology and morphology would have originated in parallel several times, as has also been reported for *Heliosperma pusilla* (Trucchi et al., 2017). This offers the opportunity to investigate whether this parallelism is also recognizable at the anatomical, physiological, and genetic levels.

## CONCLUSIONS

We provided strong evidence for the origin of regional and local endemics (*M. intermedia*, *M. markgrafii*, *M. papulosa*, possibly *M. tommasinii*) via peripatric speciation from a widespread, still extant ancestor (*M. muscosa*) in *Moehringia* sect. *Moehringia* in the European Alps. Such a mode of speciation can only be detected using multiple samples of the species, along with DNA sequence data, which allow the resolution of relationships among very closely related entities. Use of such an approach in other lineages from the Alps, other mountain ranges, or geographical settings where Quaternary glacial refugial areas were small and scattered may help specify processes of Quaternary speciation beyond the finding that they are linked to climatic change.

## AUTHOR CONTRIBUTIONS

Conceptualization: J.W.K.; formal analysis: K.M., A.M.P., D.F.M.B.; investigation: J.W.K.; resources: G.K.; supervision: J.W.K., G.K., D.F.M.B.; visualization: D.F.M.B.; Writing original draft: J.W.K., D.F.M.B.; review and editing: all authors.

## Supporting information


**Figure S1:** Geographical origin of all samples of *Moehringia* sect. *Moehringia* used in this study (also see Appendix S1: Table S2).
**Figure S2:** (A) Coalescent‐based species tree of *Moehringia* inferred with ASTRAL‐IV from 829 orthologous gene trees.
**Figure S3:** Cladogram from the coalescent‐based species tree of *Moehringia* inferred with ASTRAL‐IV from 829 orthologous gene trees.
**Figure S4:** Cladogram from the coalescent‐based species tree of *Moehringia* inferred with ASTRAL‐IV from 829 orthologous gene trees.
**Figure S5:** Phytop estimates of incomplete lineage sorting (ILS) and introgression/hybridization (IH) across the ASTRAL‐IV phylogeny of *Moehringia*.
**Figure S6:** Plot of quartets not satisfied by each of the 13 networks inferred from CAMUS for *Moehringia* sect. *Moehringia*.
**Figure S7:** Phylogenetic network of *Moehringia* sect. *Moehringia* showing four reticulation events inferred with CAMUS from 789 gene trees.
**Figure S8:** Time‐calibrated tree of *Moehringia* inferred with RelTime based on the ASTRAL‐IV coalescent‐based species tree and a filtered alignment of 75,225 bp.
**Figure S9:** Time‐calibrated tree of *Moehringia* inferred with BEAST2 based on the ASTRAL‐IV coalescent‐based species tree and 20‐locus alignment of 43,254 bp.
**Figure S10:** (A) Concatenated maximum likelihood tree of *Moehringia* inferred with IQ‐TREE from 80 plastome CDS.
**Figure S11:** Cladogram from the concatenated maximum likelihood tree of *Moehringia* inferred with IQ‐TREE from 80 plastome CDS.
**Figure S12:** Histograms of the cumulative variance from principal component analysis (PCA) of *Moehringia* sect. *Moehringia* inferred with PLINK for (A) 65 samples and 44,304 genome‐wide SNPs; (B) 61 samples and 35,825 SNPs; and (C) 58 samples and 33,468 SNPs.
**Figure S13:** Cross‐validation error estimations from ADMIXTURE runs for *K* = 2–16.
**Figure S14:** ADMIXTURE model‐based clustering analysis of all species (65 target enrichment accessions) of *Moehringia* sect. *Moehringia*, based on 44,304 genome‐wide SNPs, showing *K* = 2–16.
**Figure S15:** (A) *D*‐statistic and (B) *f*_4_ admixture ratio among members of *Moehringia* sect. *Moehringia* calculated from ABBA/BABA site patterns using 597,387 genome‐wide SNPs. Colors correspond to statistical support (light to dark) and magnitude (blue to red) of the statistics.

**Table S1:** Species sampled for nuclear tree‐based orthology for target enrichment bait design.
**Table S2:** Species and accessions sampled for target enrichment data generation.
**Table S3:** Publicly available plastid genomes used for phylogenetic analyses.
**Table S4:** Sequencing, orthologs, paralogs, and plastome assembly statistics.
**Table S5:** Species and accessions sampled for plastome haplotype network.
**Table S6:** Significant trio‐level introgression tests from Dsuite among members of *Moehringia* sect. *Moehringia*.
**Table S7:** Significant branch‐level *Zb* signals from the Dsuite *f*‐branch analysis among members of *Moehringia* sect. *Moehringia*.

## Data Availability

Transcriptome and target enrichment data generated for this study are available in the National Center for Biotechnology Information (NCBI) BioProject PRJNA1477318 (see Appendix S1: Tables S1 and S2 for SRA accession numbers). Analysis files are available from the Zenodo repository: https://doi.org/10.5281/zenodo.20643792.
