# Supplementary material for: Incomplete climate‐driven peripatric speciation in Moehringia sect. Moehringia (Caryophyllaceae) in the European Alps
Source: Am J Bot. 2026 Aug 26;113(9):e70252. doi: 10.1002/ajb2.70252 (PMC13595479; doi:10.1002/ajb2.70252)
Supplement: Supplementary file 1 [file AJB2-113-e70252-s002.pdf]

Appendix S2. Supplemental Figures S1–S15.

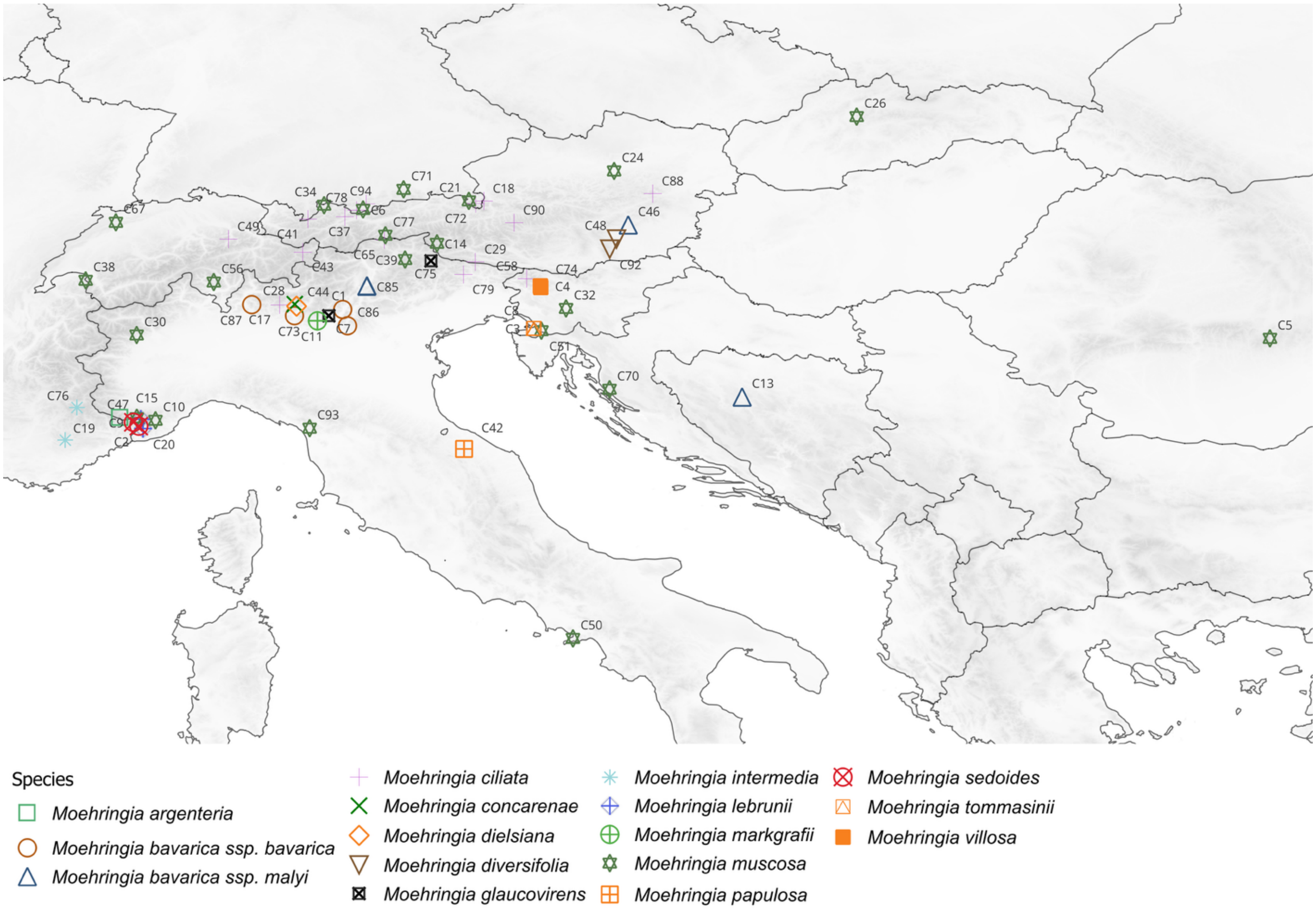

Figure S1. Geographical origin of all samples of *Moehringia* sect. *Moehringia* used in this study (also see Appendix S1: Table S2).

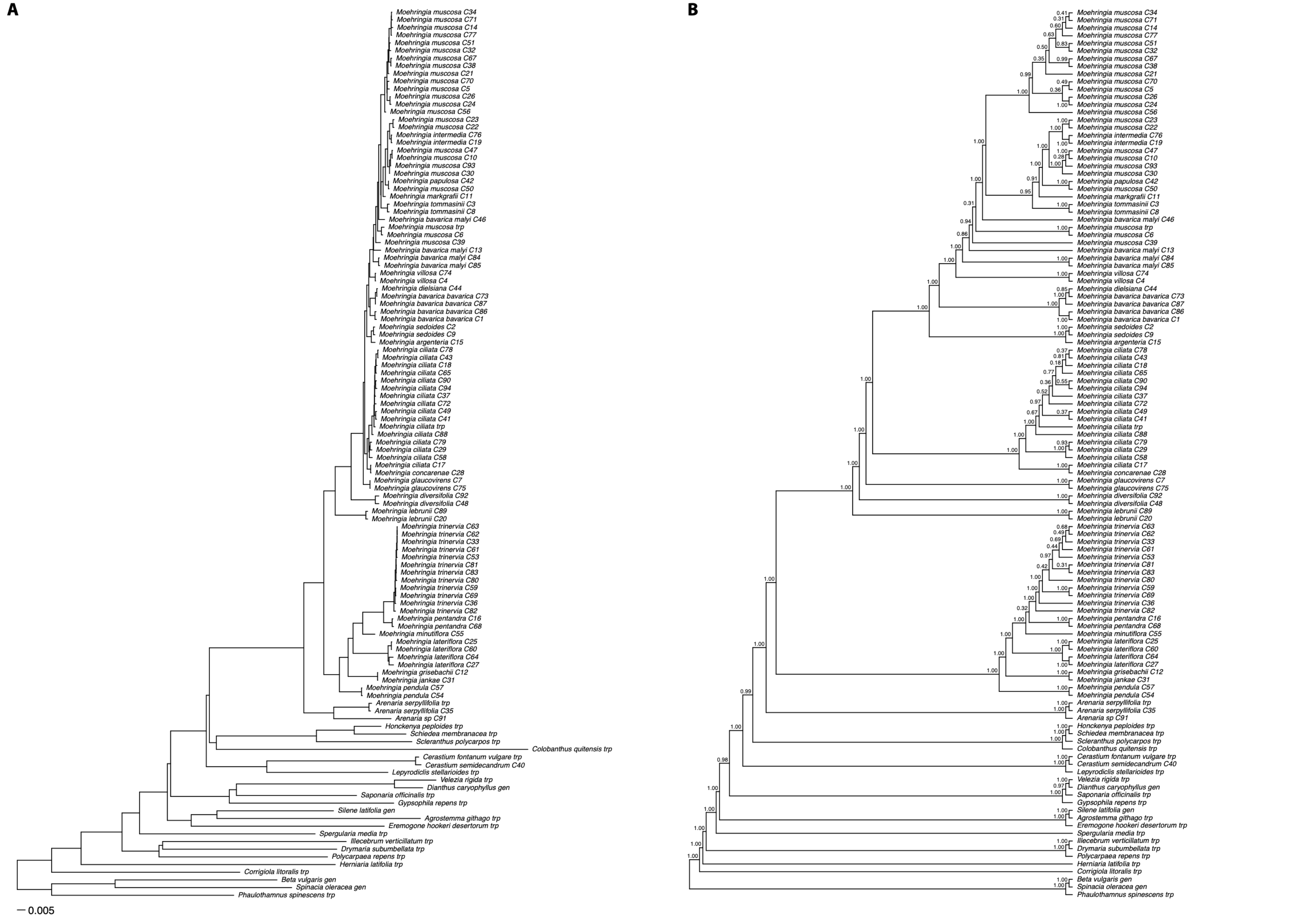

Figure S2. (A) Coalescent-based species tree of *Moehringia* inferred with ASTRAL-IV from 829 orthologous gene trees. Branch lengths in number of substitutions per site (scale bar). (B) Cladogram of A showing local posterior probability (LLP) values next to nodes.

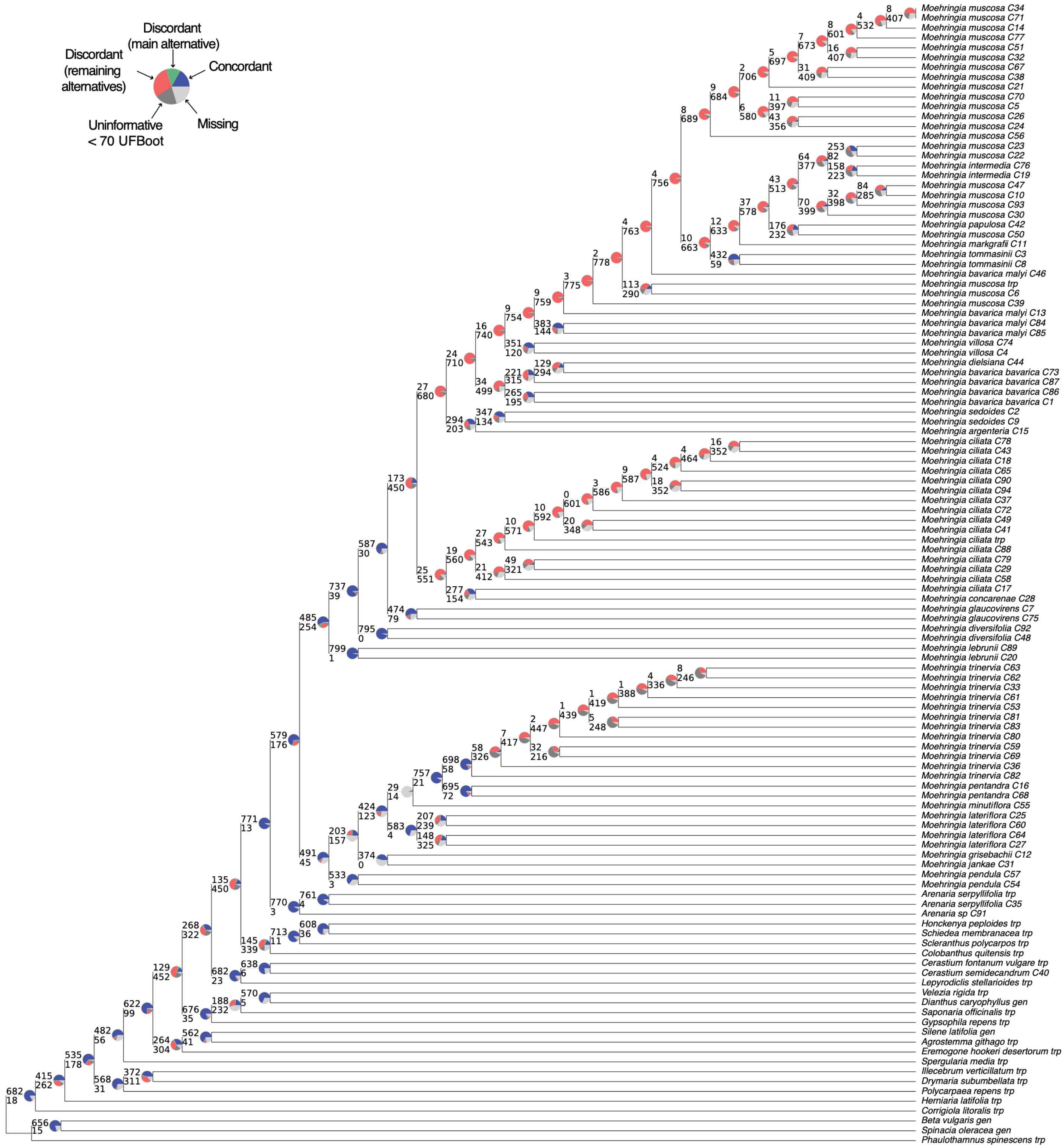

Figure S3. Cladogram from the coalescent-based species tree of *Moehringia* inferred with ASTRAL-IV from 829 orthologous gene trees. Pie charts represent the proportion of gene trees that support that clade (blue), the main alternative bifurcation (green), the remaining alternatives (red), the remaining alternatives with (conflict or support) ultrafast bootstrap (UFBoot) lower than 70% (dark gray), and missing data (light gray). Numbers above and below branches represent the number of concordant (blue in the pie charts) and discordant (red + green in the pie charts) gene trees, respectively.

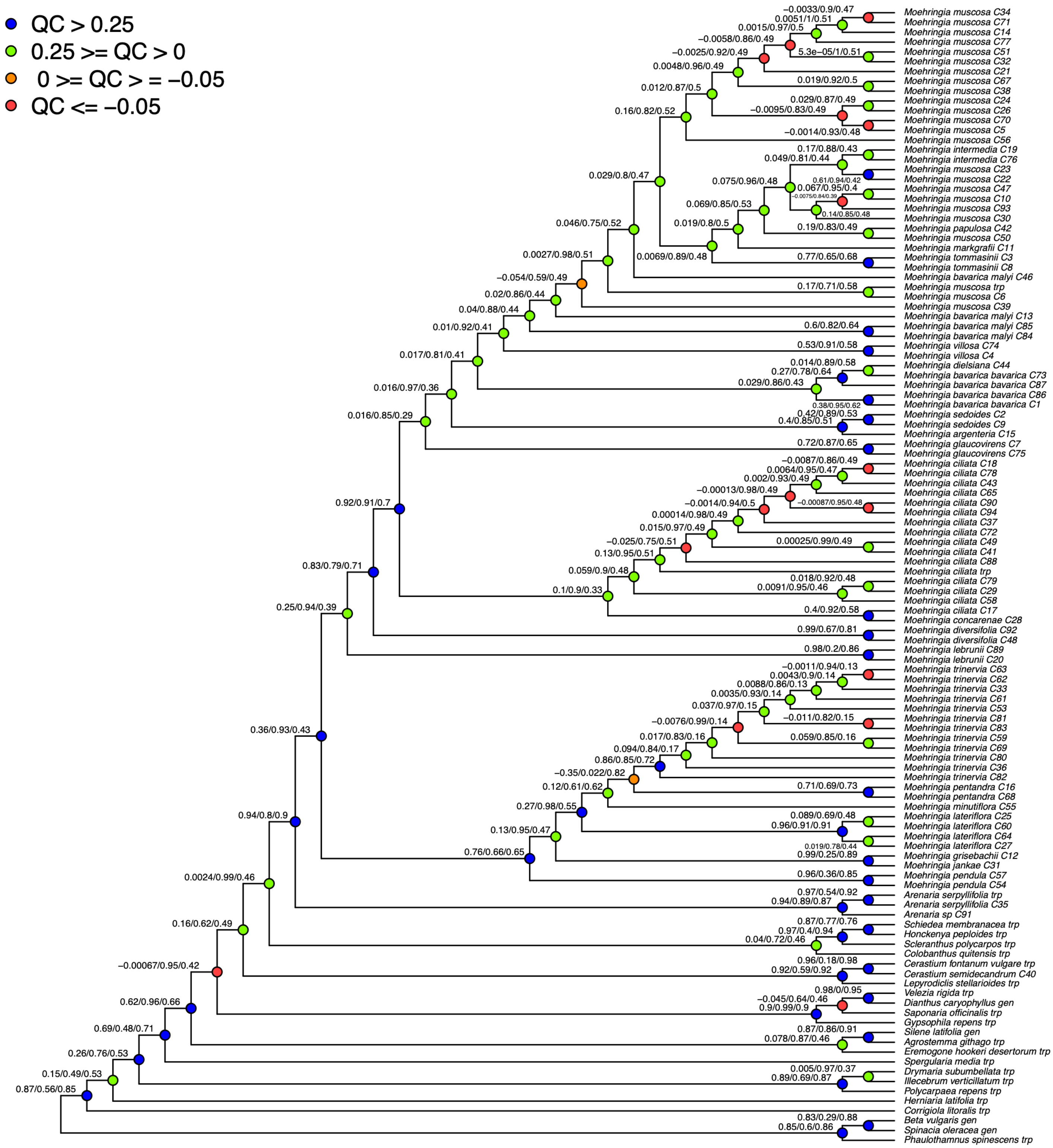

Figure S4. Cladogram from the coalescent-based species tree of *Moehringia* inferred with ASTRAL-IV from 829 orthologous gene trees. Quartet sampling (QS) scores are shown next to each node: quartet concordance/quartet differential/quartet informativeness. Colored circles on the nodes represent QS score ranges.

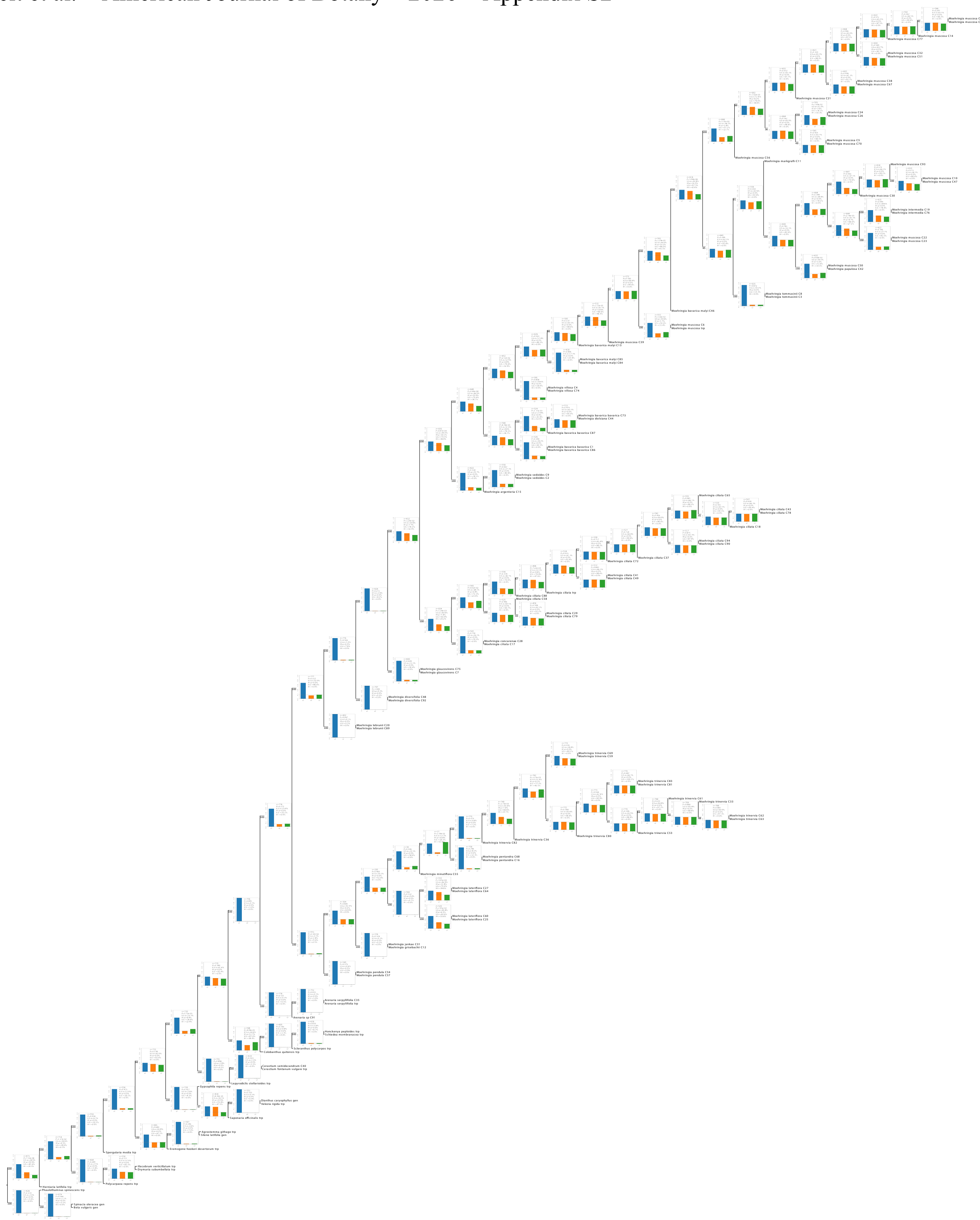

Figure S5. Phytop estimates of incomplete lineage sorting (ILS) and introgression/hybridization (IH) across the ASTRAL-IV phylogeny of *Moehringia*. Histograms at each node represent the quartet frequencies (q1, q2, q3) from ASTRAL. Shown on top of the histograms are: *n*, representing the number of gene trees; *P*, showing the *P*-value of the χ^2 test to check whether the frequencies of q2 and q3 are equal; ILS-i and IH-I, representing the calculated ILS (scaled 0–100%) and IH (scaled 0–50%) indices, respectively; ILS-e and IH-e, representing the proportion of gene tree topological incongruence that can be explained by ILS (q2 ≈ q3) and IH (q2 ≠ q3), respectively.

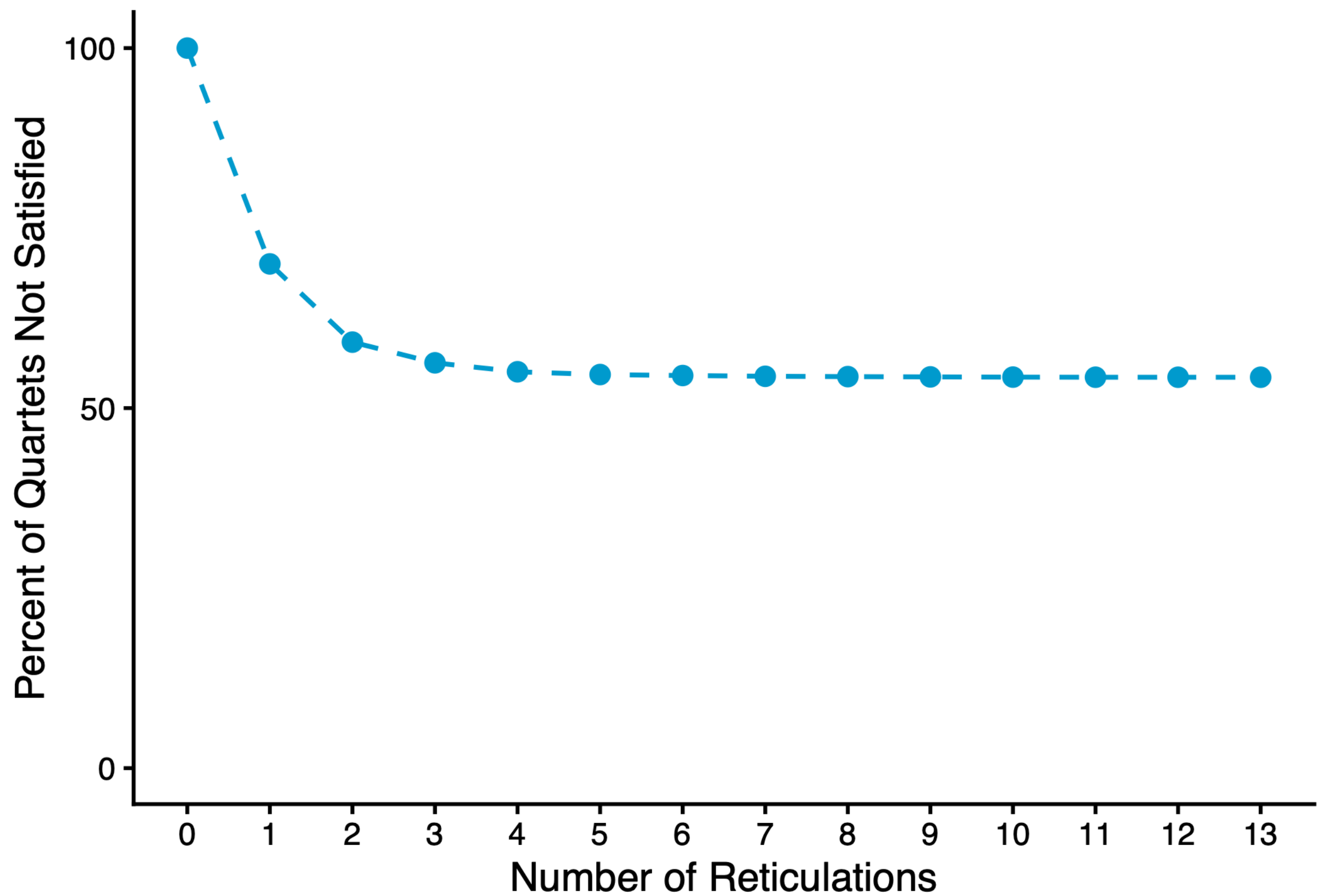

Figure S6. Plot of quartets not satisfied by each of the 13 networks inferred from CAMUS for *Moehringia* sect. *Moehringia.*

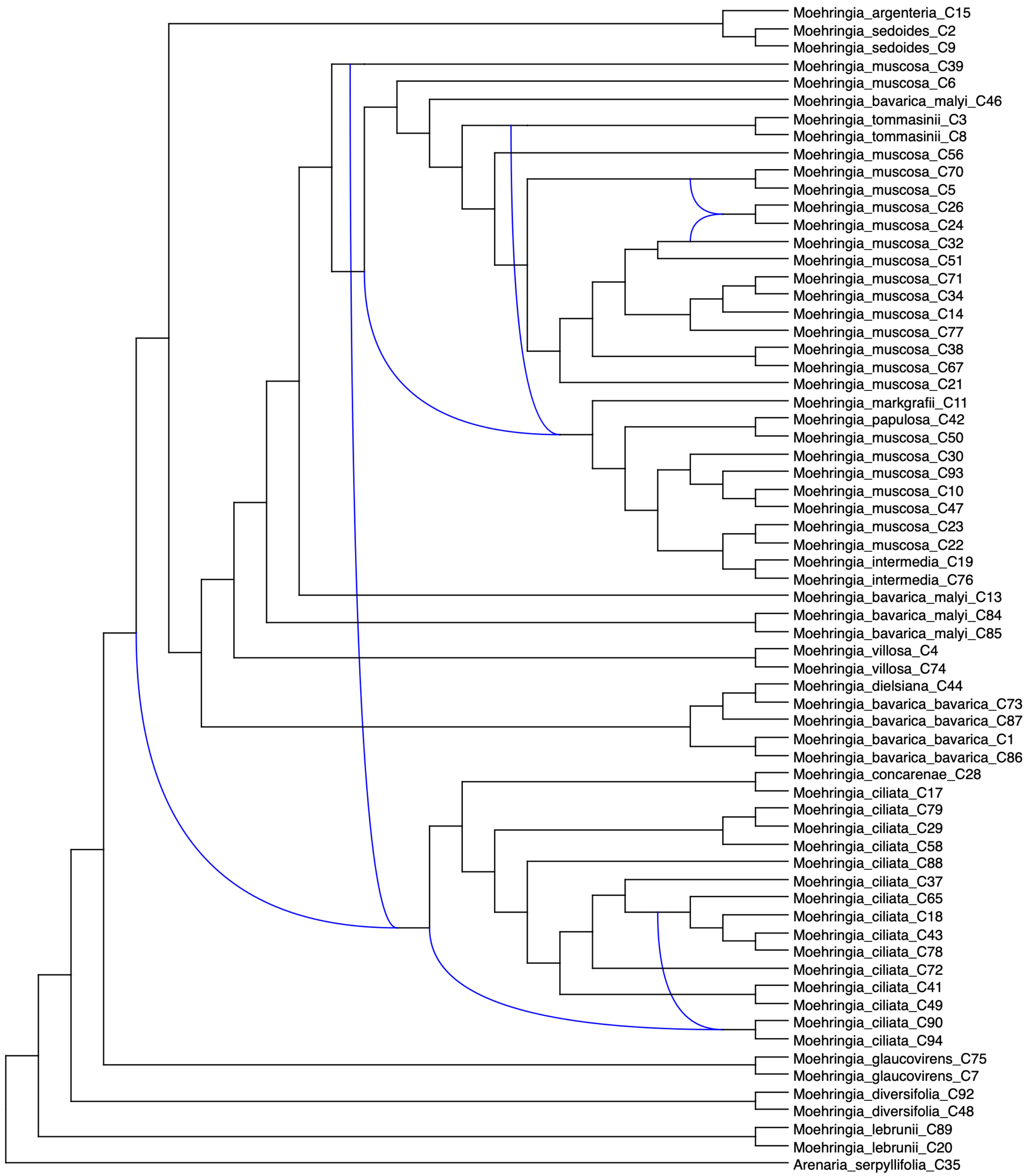

Figure S7. Phylogenetic network of *Moehringia* sect. *Moehringia* showing four reticulation events inferred with CAMUS from 789 gene trees.

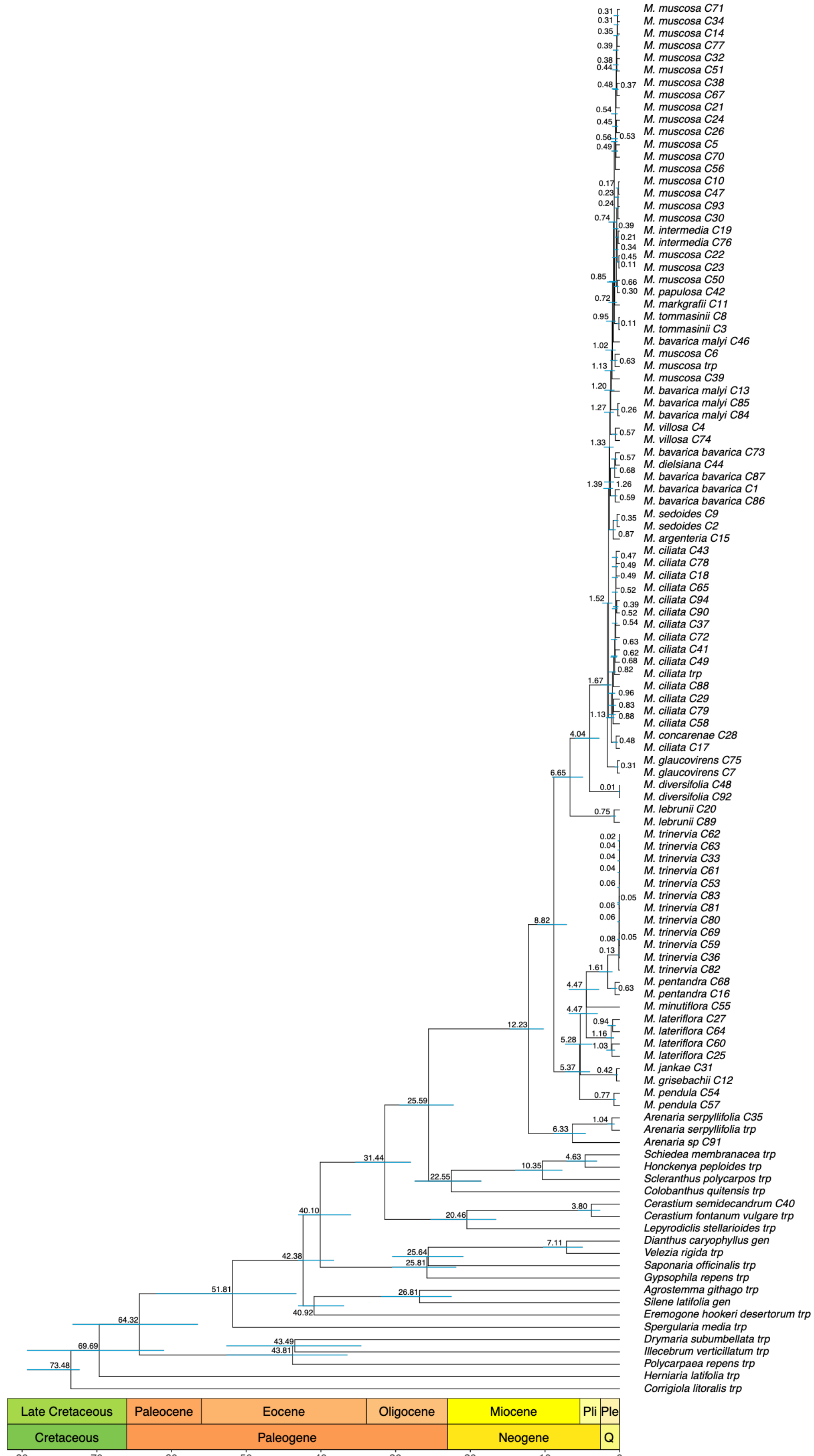

Figure S8. Time-calibrated tree of *Moehringia* inferred with RelTime based on the ASTRAL-IV coalescent-based species tree and a filtered alignment of 75,225 bp. Node labels represent absolute times of divergence, and blue bars represent the 95% confidence intervals (CI). The time unit at the bottom of the geological time scale is million years.

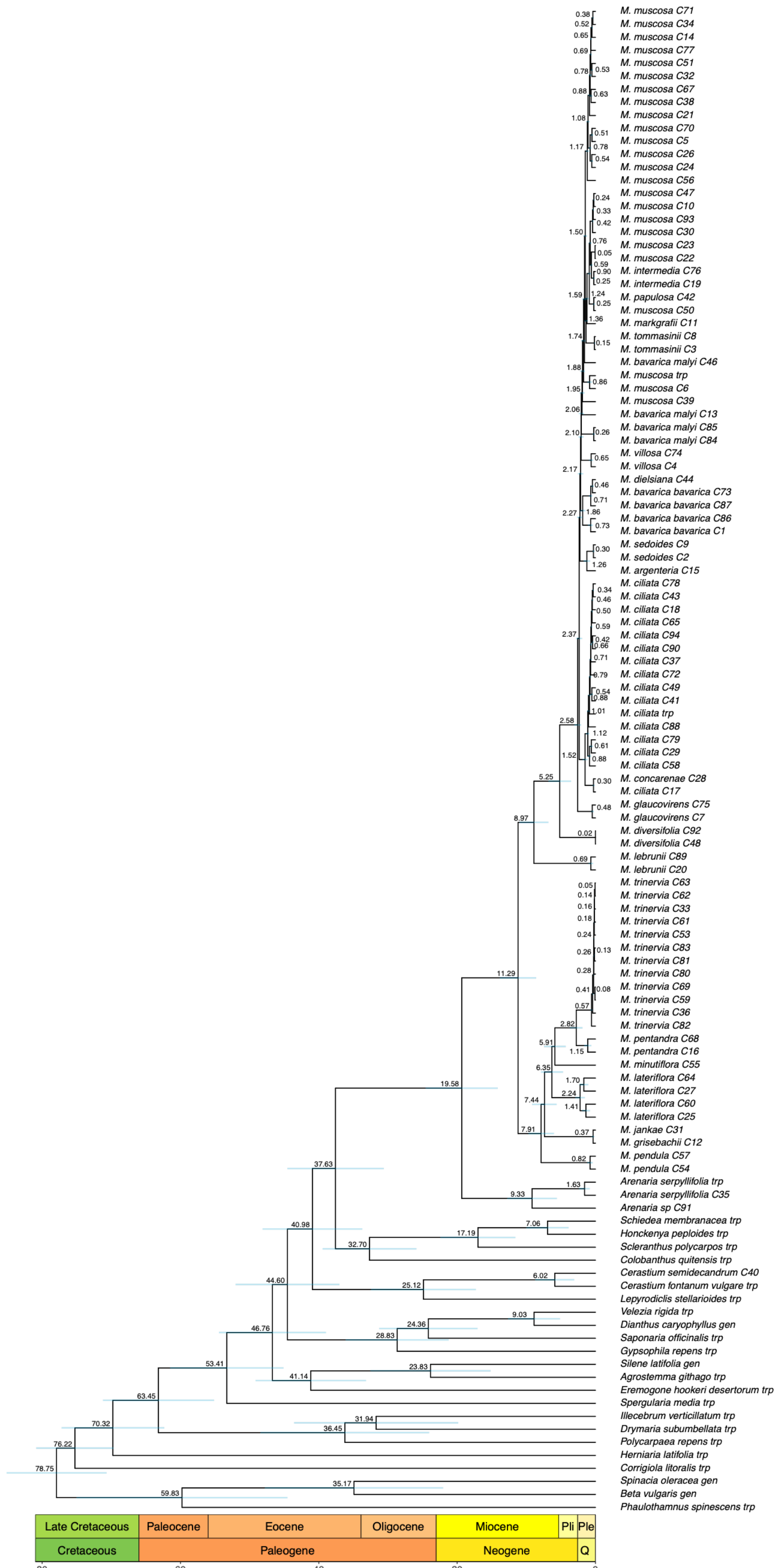

Figure S9. Time-calibrated tree of *Moehringia* inferred with BEAST2 based on the ASTRAL-IV coalescent-based species tree and 20-locus alignment of 43,254 bp. Node labels represent absolute times of divergence, and blue bars represent the 95% highest posterior density (HPD) intervals. The time unit at the bottom of the geological time scale is million years.

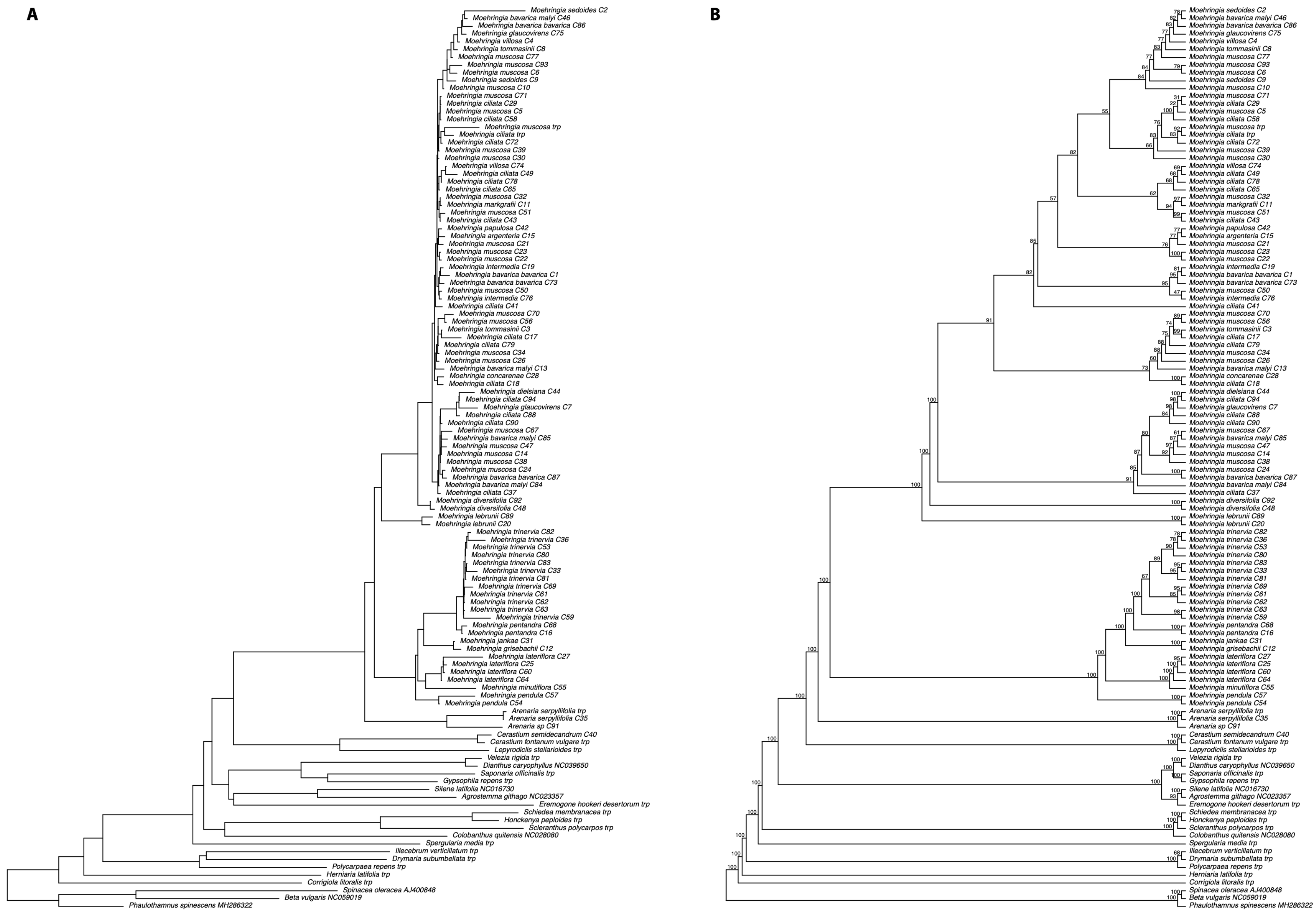

Figure S10. (A) Concatenated maximum likelihood tree of *Moehringia* inferred with IQ-TREE from 80 plastome CDS. Branch lengths in number of substitutions per site (scale bar). (B) Cladogram of panel A showing Ultrafast bootstrap (UFBoot) values next to nodes.

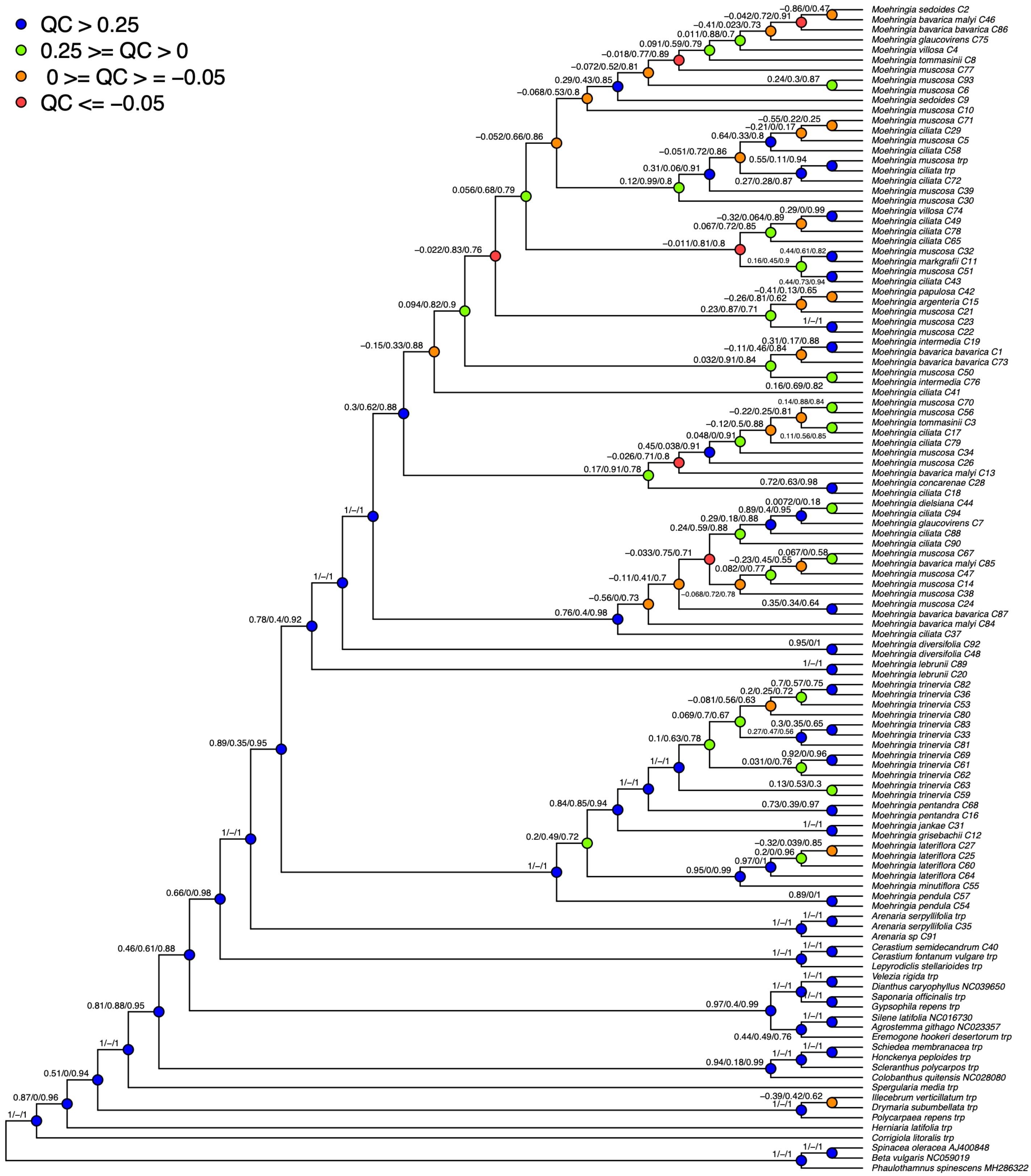

Figure S11. Cladogram from the concatenated maximum likelihood tree of *Moehringia* inferred with IQ-TREE from 80 plastome CDS. Quartet sampling (QS) scores are shown next to each node: quartet concordance/quartet differential/quartet informativeness. Colored circles on the nodes represent QS score ranges.

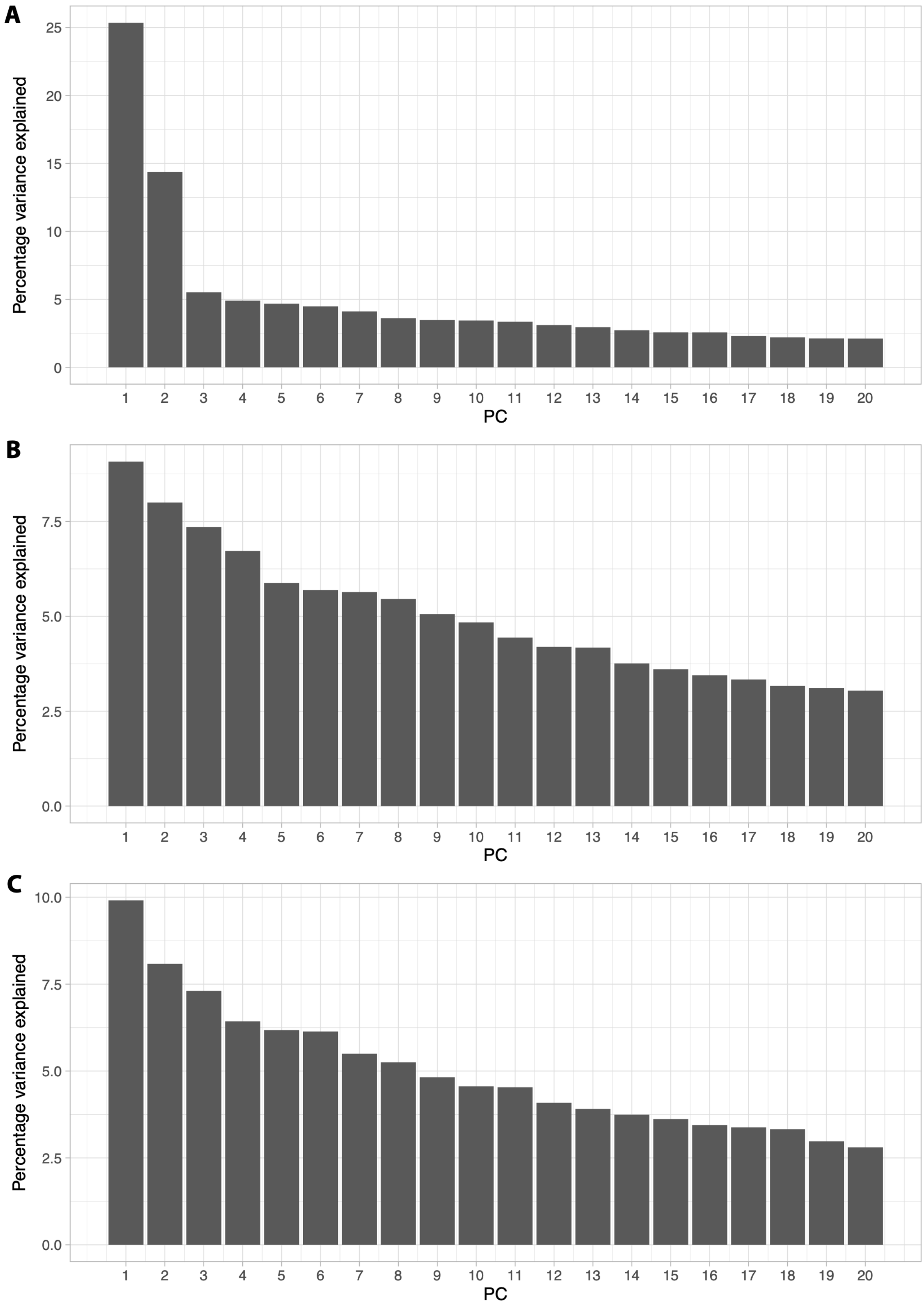

Figure S12. Histograms of the cumulative variance from principal component analysis (PCA) of *Moehringia* sect. *Moehringia* inferred with PLINK for (A) 65 samples and 44,304 genome-wide SNPs; (B) 61 samples and 35,825 SNPs; and (C) 58 samples and 33,468 SNPs.

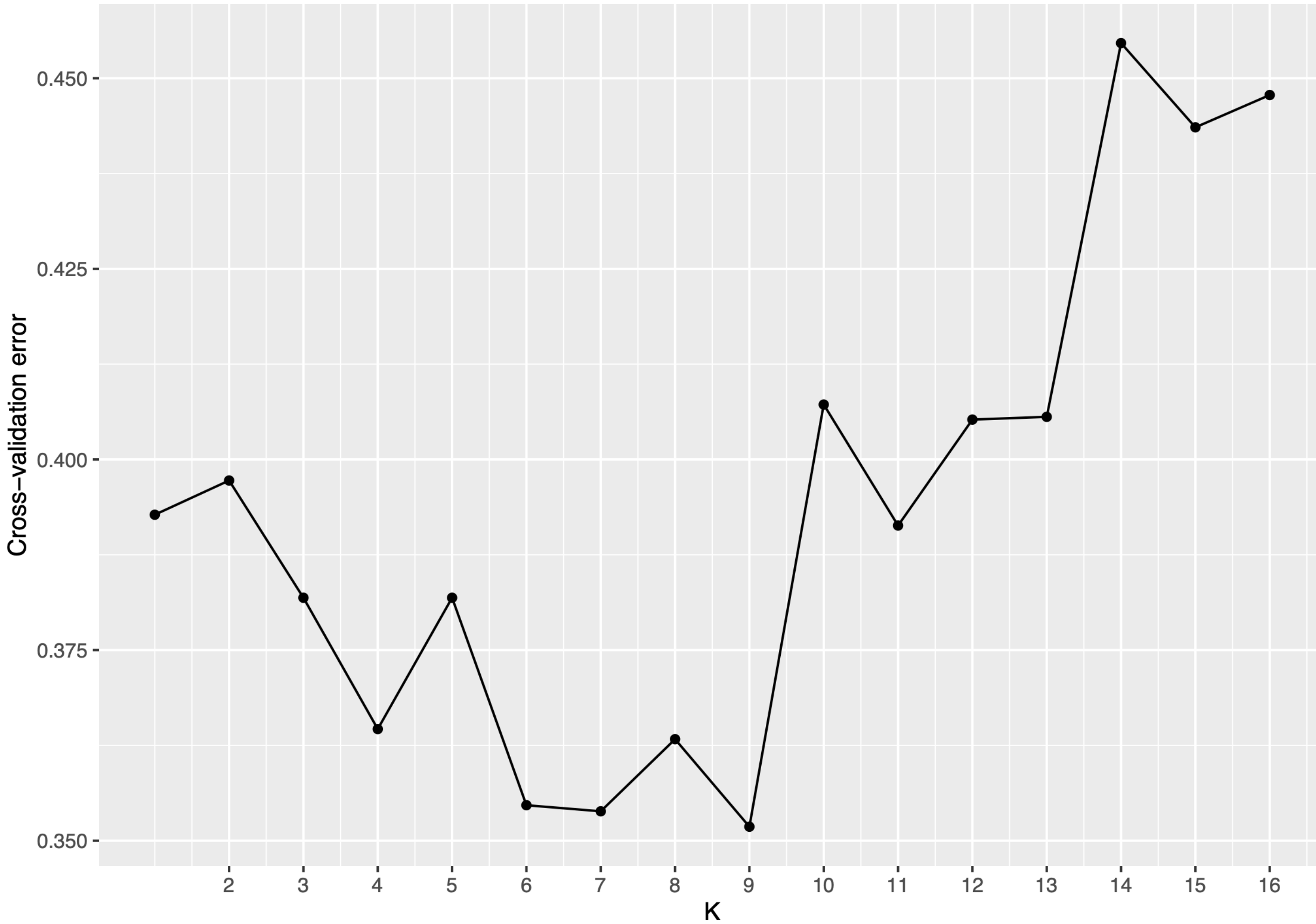

Figure S13. Cross-validation error estimations from ADMIXTURE runs for *K* = 2–16.

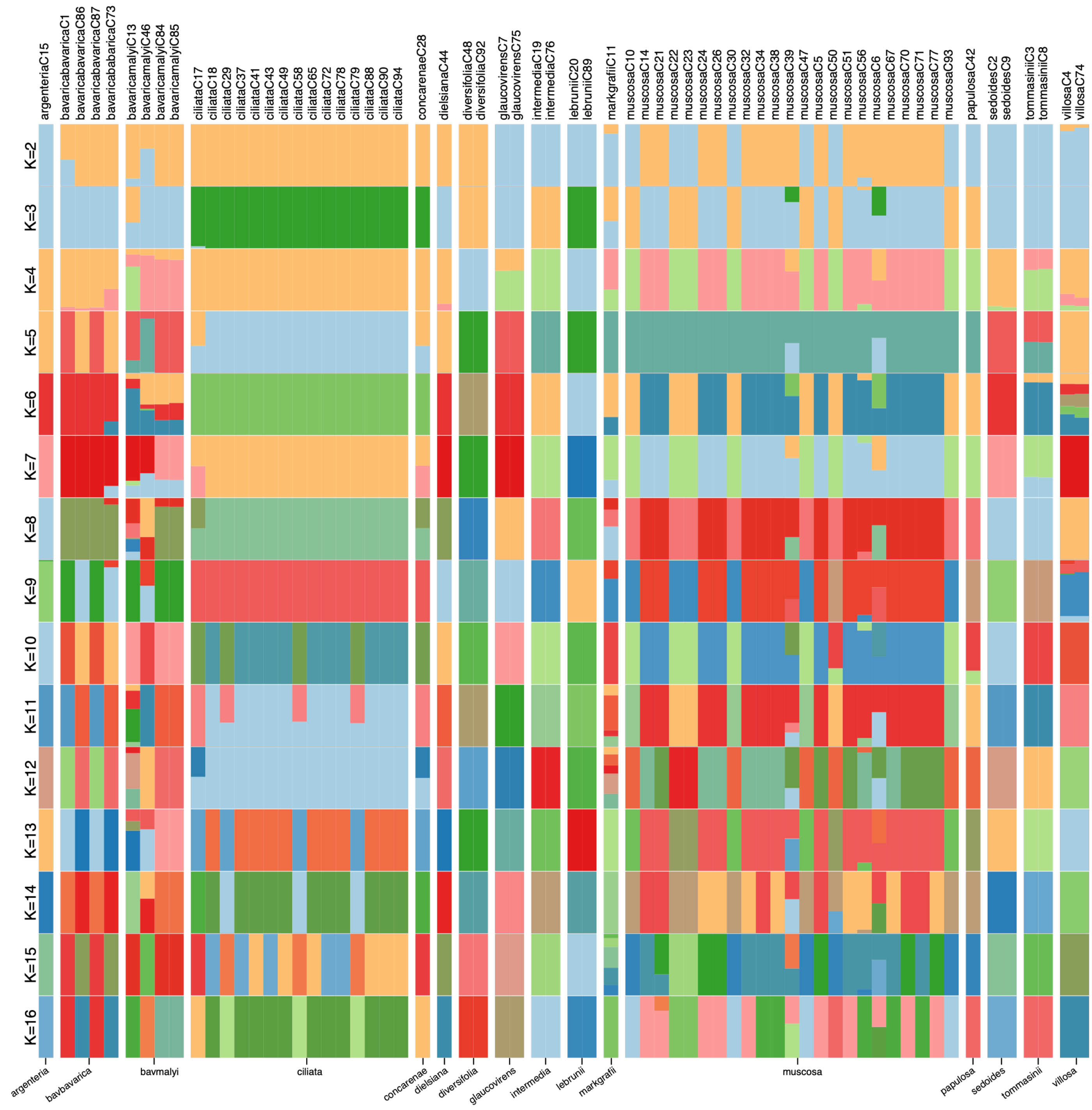

Figure S14. ADMIXTURE model-based clustering analysis of all species (65 target enrichment accessions) of *Moehringia* sect. *Moehringia,* based on 44,304 genome-wide SNPs, showing K = 2–16.

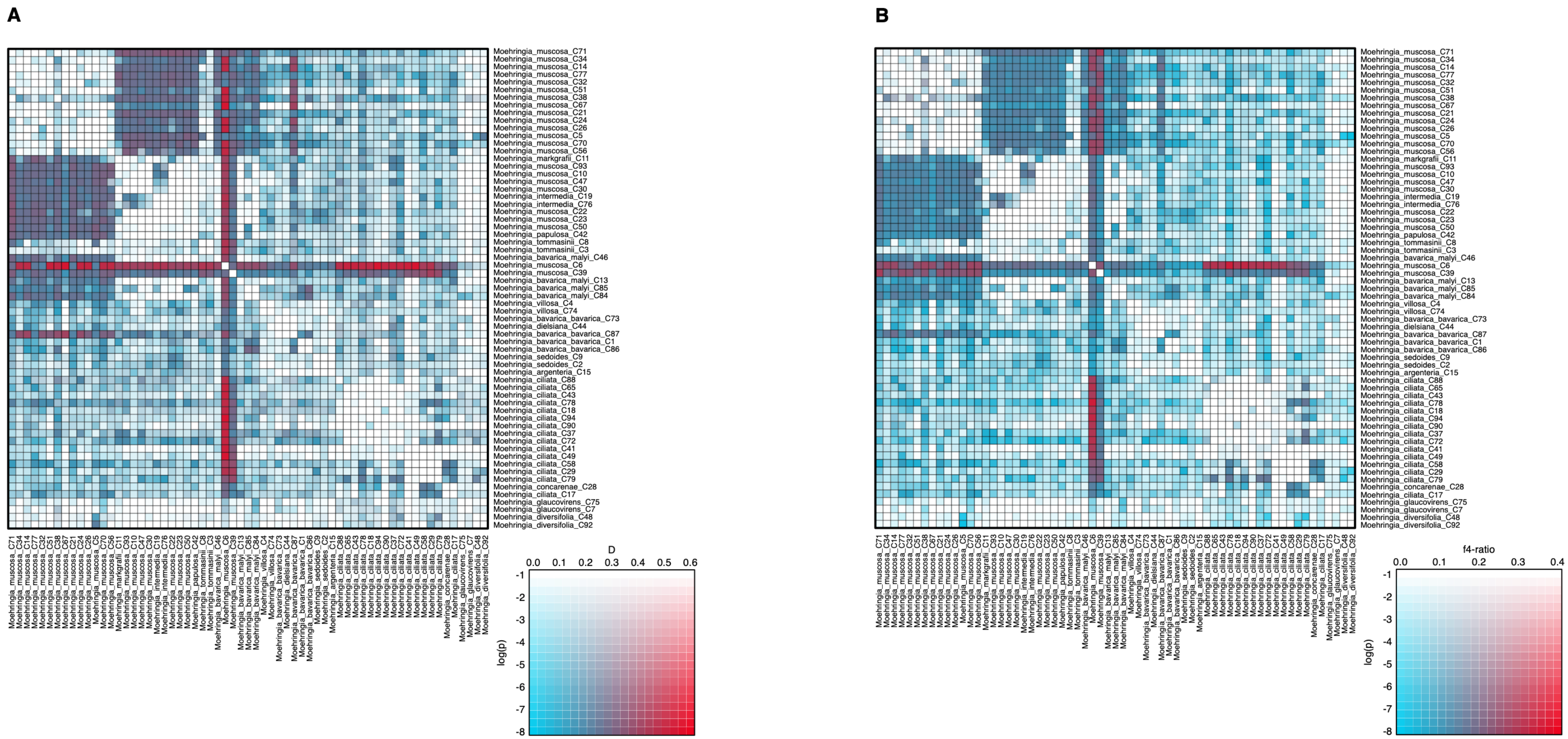

Figure S15. (A) *D*-statistic and (B) f_4 admixture ratio among members of *Moehringia* sect. *Moehringia* calculated from ABBA/BABA site patterns using 597,387 genome-wide SNPs. Colors correspond to statistical support (light to dark) and magnitude (blue to red) of the statistics.
